# Tissue-specific gene dosage disruption is a key feature and pathogenic mechanism of structural variants in the human genome

**DOI:** 10.1186/s13073-026-01653-7

**Published:** 2026-04-18

**Authors:** Xubing Liu, Zhao Chen, Qian Jiang, Haoyu Shen, Hongyu Liu, Zhenguo Wang, Zhe Li, Jihong Wu, Hong Jiang, Xin Li

**Affiliations:** 1 Department of Neurology, Xiangya Hospital, Central South University, Changsha, People’s Republic of China; 2 Shanghai Institute of Nutrition and Health, University of Chinese Academy of Sciences, Chinese Academy of Sciences, Shanghai, China; 3 Eye Institute and Department of Ophthalmology, Eye & ENT Hospital, Fudan University, Shanghai, China; 4 NHC Key Laboratory of Myopia, Key Laboratory of Myopia, Fudan University), Chinese Academy of Medical Sciences, Shanghai, China; 5 Shanghai Key Laboratory of Visual Impairment and Restoration, Shanghai, China; 6 Key Laboratory of Hunan Province in Neurodegenerative Disorders, Central South University, Changsha, People’s Republic of China; 7 National Clinical Research Center for Geriatric Diseases, Central South University, Changsha, People’s Republic of China; 8 National International Collaborative Research Center for Medical Metabolomics, Central South University, Changsha, People’s Republic of China; 9 Department of Neurology, The Third Xiangya Hospital, Central South University, Changsha, People’s Republic of China

**Keywords:** Structural variants, Tissue-specific dosage disruption, Transcript disruption ratio, Transcriptome-aware variant interpretation, Gene dosage, Inherited retinal disease, Hereditary ataxia, Neurodegenerative disorders

## Abstract

**Background:**

Structural variants (SVs) are important contributors to genetic disorders, yet their effects on gene dosage, particularly in a tissue-specific context, remain underexplored.

**Methods:**

Leveraging large-scale transcriptomic data, we analyzed the tissue specific dosage disrupting effect of SVs in the human genome. To characterize this tissue-specific dosage effect, we developed a computational framework, PathoSV, which captures this impact via a Transcript Disruption Ratio (TDR). We further evaluated these tissue-specific dosage effects in patients with inherited neurodegenerative diseases.

**Results:**

We find that dosage disruption is a key feature of rare structural variants and such effects are largely tissue specific. Further identifying such tissue-specific dosage disrupting SVs among 530 previously unresolved individuals with inherited retinal disease (IRD) or hereditary ataxia (HA), we made a molecular diagnosis in 10.1% and 3.2% of cases, respectively. We identified pathogenic dosage-disrupting SVs in critical, tissue-specific genes such as *EYS* in the retina and *ITPR1* and *SPAST* in the cerebellum.

**Conclusions:**

We show that tissue-specific disruption of gene dosage is a characteristic functional consequence of rare SVs in the human genome and an important contributor to pathogenicity. Quantifying transcript-level dosage disruption clarifies the transcriptomic context in which pathogenic effects arise in complex neurodegenerative disorders and suggests a broader role for tissue-specific dosage disruption in disease. PathoSV is available at: https://github.com/xlilab/PathoSV.

**Supplementary Information:**

The online version contains supplementary material available at 10.1186/s13073-026-01653-7.

## Background

Structural variants (SVs), encompassing deletions, duplications, insertions, inversions, and translocations, are integral to human genetic diversity and recognized as contributors to disease [1, 2]. While accurately identifying SVs remains technically challenging, often confounded by their size and complexity with short-read sequencing [3], a greater barrier to understanding their role in disease lies in their functional interpretation. SVs are a major source of differences in human gene expression [4, 5] and account for a substantial fraction of rare protein-truncating events [1]. However, their contribution is often underestimated, hindering accurate diagnosis and genetic counseling [6].

The pathogenicity of an SV is tied to its effect on gene dosage, and emerging evidence suggests that functional interpretation must be "transcriptome-aware" [2, 7, 8]. We have previously identified cross-tissue effects of SVs [2, 4], however, their tissue-specific effects remain underexplored. Due to complex patterns of alternative splicing, a single gene can produce numerous transcript isoforms, each with a unique expression profile across different tissues. Consequently, a variant's functional consequence depends on which specific transcripts it disrupts within the relevant tissue context. Incorporating transcript expression data has been shown to effectively filter misleading annotations and better capture true functional effects [8].

This need for a transcriptome-aware, dosage-focused evaluation is particularly relevant for neurodegenerative disorders [9, 10], especially those with a major genetic basis such as inherited retinal diseases (IRDs) [11] and hereditary ataxias (HAs) [12]. These conditions arise from dysfunction in highly specialized neural tissues, such as the retina and cerebellum, which have uniquely regulated transcriptomic landscapes. Genetic variations implicated in these diseases may exert their effects by modulating the expression of specific isoforms critical for neuronal function [12, 13]. The nervous system appears particularly vulnerable to perturbations in gene dosage [14], as its high-order functions depend on systems with minimal redundancy, such as the signal amplification cascades in phototransduction and the precarious energetic balance maintained by mitochondrial networks. This inherent dosage sensitivity suggests that structural variants (SVs), potent modulators of gene dosage, may be a significant, yet poorly understood, driver of neurodegenerative disorders. Based on an RNA-informed map of dosage sensitivity, we previously observed that dosage sensitivity is an intrinsic, tissue-specific property of genes, tightly linked to their functional role [15]. One finding was the divergence in constraint between different functional classes: "signaling" genes, including transcription factors, protein kinases, and ion channels, were found to be highly dosage-sensitive, whereas "effector" genes were generally dosage-resilient. In particular, the dosage sensitivity map suggests that the nervous system is particularly enriched with these dosage-sensitive genes, with ion channels, for example, showing strong dosage constraint specifically in the brain.

Leveraging large-scale transcriptomic data, we systematically evaluated tissue-specific dosage effects of structural variants (SVs). Our analyses suggest that tissue-specific dosage disruption is a key feature of SVs in the human genome. While dosage-disrupting effects caused by SVs in disease-relevant tissues are rare in the population, they represent a significant pathogenic mechanism in genetic disorders, particularly neurodegenerative diseases.

## Methods

### Study cohorts and population reference datasets

This study involved several distinct human cohorts. To construct a harmonized background database for allele frequency (AF) estimation, raw sequencing data from two large-scale population cohorts were re-processed using the same pipeline applied to the patient samples. The 1KGP East Asian cohort included 196 individuals (East Asian ancestries: CHS + CHB) from the 1 KG Project (~ 30 × WGS). Data were obtained from the 1KGP website (https://www.internationalgenome.org/) [16]. The GTEx European cohort included 838 individuals (mainly of European ancestry) from the Genotype-Tissue Expression (GTEx) v8 project [17]. Data were obtained from dbGaP (accession phs000424.v8.p2) and the GTEx portal (https://www.gtexportal.org/home/) [18]. The Inherited Retinal Disease (IRD) cohort included 218 unrelated Chinese patients with IRDs who remained genetically unresolved after initial panel sequencing (total *n* = 646 including family members); the recruitment, ethical approval, and clinical characterization of this cohort were previously described [19]. The Hereditary Ataxia (HA) cohort included 312 unrelated Chinese patients presenting with ataxia symptoms and previously negative for known pathogenic SNVs and repeat expansions.

### Whole genome sequencing and SNV/Indel calling

WGS was performed on the IRD and HA cohorts. Raw sequence data (Fastq) for all cohorts underwent quality control using FastQC (v0.11.9; https://www.bioinformatics.babraham.ac.uk/projects/fastqc/). Reads were aligned to the GRCh38/hg38 human reference genome using the Burrows-Wheeler Aligner (BWA-MEM v0.7.17) [20]. Post-alignment processing included marking duplicate reads using Picard MarkDuplicates (v2.25.0; Broad Institute, http://broadinstitute.github.io/picard/) and base Quality Score Recalibration (BQSR) using the Genome Analysis Toolkit (GATK v4.2.6.1) [21]. BAM file quality was assessed, requiring mean coverage > 15x, chimeric reads < 0.05%, normal insert size distribution (Picard CollectInsertSizeMetrics), and contamination rate < 5% (VerifyBamID2 v1.1.3 [22]).

SNVs and indels were identified using the GATK best practices workflow. GATK HaplotypeCaller (v4.2.6.1) generated per-sample GVCFs, which were combined (CombineGVCFs) and jointly genotyped (GenotypeGVCFs). Variant Quality Score Recalibration (VQSR) was applied. Variants failing VQSR (sensitivity < 99.8%) or meeting filtering criteria (Low Complexity Regions, Inbreeding Coefficient < −0.3, Hardy–Weinberg *p* < 1e-6) were removed using GATK and Hail (v0.2; https://hail.is). Low-confidence genotypes were filtered based on DP (< 10), GQ (< 25), and Allelic Balance (AB). Variants were annotated using gnomAD v3.0 [23] for allele frequency. Ensembl VEP (v108) [24] annotated variants against Gencode v26 [25]. Pathogenicity prediction tools (CADD v1.6 [26]; SpliceAI [27]; AlphaMissense [28]; LOFTEE [23]) and ClinVar [29] were used for prioritization based on established criteria (e.g., SpliceAI > 0.5, CADD > 20). Repeat expansions in the Ataxia cohort were assessed using ExpansionHunter (v4.0.2) [30].

### Structural variant calling and frequency estimation

Because different algorithms are optimized for different SV types and sizes, we used an integrated approach combining multiple callers and a rigorous filtering strategy (Additional file 1: Fig. S1, Table S1). Manta (v1.6.0) [31] was used for per-sample SV calling as the primary candidate generation step, utilizing paired-end and split-read evidence for breakpoint precision. BND calls were converted to INV format. Lumpy (v0.2.13) [32], integrating read-pair, split-read, and read-depth signals (run via SpeedSeq pipeline [33]), and CNVnator (v0.4.1) [34], a read-depth based CNV caller (run with 150 bp window size), were also utilized, primarily to provide supporting evidence within the filtering workflow.

Calls in problematic genomic regions (HLA, decoys, high CN; SpeedSeq annotations [33]) were excluded. To enhance specificity while maintaining sensitivity, Manta SV calls were subjected to a filtering workflow. Short SVs (< 200 bp) were re-genotyped using Paragraph (v2.4a) [35]. Only SVs with a confident genotype call (HET or HOM) were retained. Medium CNVs (200 bp ≤ length < 100 kb, DEL/DUP) required supporting evidence (> 80% reciprocal overlap) from either Lumpy or CNVnator calls. This approach leveraged complementary signal types (RD + RP/SR) for improved confidence. Long CNVs (length ≥ 100 kb, DEL/DUP) required supporting evidence (> 80% reciprocal overlap) from CNVnator, considering the robustness of RD methods for large events. Other SVs (INV, INS ≥ 200 bp) were not subjected to this overlap-based filtering due to challenges in reliable validation without potential loss of true positives. This filtering workflow was implemented as a Python script using Manta/Paragraph VCFs and BED files derived from Lumpy/CNVnator outputs (via bcftools v1.10 [36]), the pipeline is available on GitHub: https://github.com/xlilab/PathoSV [37]. The same SV calling and filtering pipeline was applied to the 1KGP East Asian and GTEx European cohorts. Filtered VCFs from each population were merged using Jasmine (v1.1.5; http://github.com/mkirsche/Jasmine) [38], consolidating SVs of the same type with > 80% reciprocal overlap. Population-specific AFs were calculated, and a final background AF was assigned to each unique SV by taking the maximum AF across all populations. Rare SVs were defined as having this combined background AF < 0.01.

### The PathoSV framework for tissue-specific dosage disruption quantification

To prioritize SVs based on potential functional impact, particularly considering tissue-specific effects, we developed PathoSV. PathoSV calculates, for a given SV-gene pair and a target tissue, the proportion of the gene's total expression (TPM) contributed by transcripts whose coding exons are overlapped/disrupted by the SV. We define this metric as the Transcript Disruption Ratio (TDR), which is formulated as:

$${TDR}_{\mathrm{gene},\mathrm{tissue}}=\frac{\sum {TPM}_{\mathrm{disrupted}\_\mathrm{transcripts}}}{\sum {TPM}_{\mathrm{all}\_\mathrm{transcripts}}}.$$

Transcript annotations were derived from Gencode v26 [25]. Tissue-specific transcript TPMs were sourced from GTEx v8 [17] and normal retina RNA-seq data (*n* = 120 samples) [39]. To mitigate quantification ambiguity inherent in unstranded short-read data, the TDR calculation incorporates all annotated transcripts, comprising both protein-coding and non-coding isoforms (Additional file 1: Fig. S2). Protein-coding transcripts typically constitute the majority of gene expression (ratio > 0.5 for ~ 87% of genes; Additional file 1: Fig. S2).

PathoSV further annotates SVs with population frequency (from the database described above), gene constraint scores (from gnomAD v3.0 [23]), dosage sensitivity metric (MoDs [15]), disease association (OMIM [40]), and known pathogenicity (ClinVar [29]). A TDR > 0.25 was used as an empirical threshold for potentially pathogenic SVs. PathoSV is implemented as a Python tool, available on GitHub: https://github.com/xlilab/PathoSV [37]. Additionally, a user-friendly web interface for running PathoSV online is available at https://www.biosino.org/pathosv/ [41]. This web server enhances interpretation by incorporating a retrieval-augmented generation (RAG) approach integrated with the DeepSeek [42] large language model (LLM). This feature leverages pre-trained models synthesizing existing literature knowledge to evaluate potential relationships between a specific SV, affected gene, and phenotype.

### Pathogenic variant identification in the IRD and HA cohorts

The study focused on patient cohorts that remained genetically unresolved after previous analyses. These cohorts had already been thoroughly screened for pathogenic single nucleotide variants (SNVs) and indels using standard methods, including VEP, SpliceAI, and CADD scores, as well as ClinVar/OMIM data and inheritance patterns. Any patient with a diagnosis attributable to these small variants was already excluded.

For SVs, PathoSV was used to calculate a tissue-specific Transcript Disruption Ratio (TDR). Candidate SVs were selected if they exhibited a TDR > 0.25, using retina-specific expression data for the IRD cohort and GTEx Cerebellum expression data for the HA cohort. Variants were prioritized based on their overlap with curated, disease-specific gene lists (from OMIM/HPO/literature). For the IRD cohort, variants were filtered against a list of 792 known ocular disease genes, whereas for the HA cohort, a list of 1135 known and candidate ataxia-associated genes was used. The dosage sensitivity of the affected gene was assessed, considering the gene's established mode of inheritance in the OMIM database and its quantitative, tissue-specific tolerance score from the Map of Dosage sensitivity (MoDs). The top candidate pathogenic SVs were manually reviewed using the Integrative Genomics Viewer (IGV) [43]. Their pathogenic role was further assessed by correlating the genetic findings with the patient's specific clinical phenotype.

### Transcriptomic outlier analysis and confounder correction

To identify individuals with extreme gene expression levels (i.e., gene expression outliers), we analyzed multi-tissue RNA sequencing data from the Genotype-Tissue Expression (GTEx) project. The detailed methodology for data normalization, batch effect correction, and outlier identification has been described in previous publications [2, 7, 15]. The key steps are briefly summarized as follows. First, gene expression levels in each tissue were log-transformed. Then, to correct for potential technical and environmental confounders, we used Probabilistic Estimation of Expression Residuals (PEER) [44] to identify hidden factors. The effects of these factors, along with known covariates like genotype principal components and sex, were subsequently regressed out of the data to obtain expression residuals. These residuals were standardized for each gene to generate Z-scores for each individual, per gene, per tissue. Finally, outliers were identified based on these Z-scores; for example, an individual could be defined as a single-tissue outlier if their absolute Z-score exceeded 2, or as a multi-tissue outlier if the absolute value of their median Z-score across tissues was greater than 2.

### Tissue-specific quantification of gene dosage sensitivity

A gene's dosage sensitivity determines whether it is associated with dominant (sensitive) or recessive (tolerant) modes of inheritance, as cataloged in resources like OMIM. To systematically quantify this property, we previously developed the Map of Dosage sensitivity (MoDs) [15], a comprehensive, tissue-specific metric. MoDs integrates DNA-level evidence, such as the depletion of loss-of-function (LoF) variants in population data, with RNA-level information, like the effect sizes of expression-altering variants (eQTLs), using a machine learning model. The final MoDs score is a percentile rank for each gene in each tissue, ranging from 0 (most constrained/sensitive) to 1 (most tolerant/resilient). An empirical threshold of < 0.3 is recommended to identify dosage-sensitive genes. If a gene's score is below this threshold in any given tissue, it is likely haploinsufficient, and that tissue is a potential site of pathology for a dominant disorder. Conversely, genes with scores > 0.3 in all tissues are likely haplosufficient and associated with recessive conditions.

## Results

### Overview of the PathoSV framework for quantifying tissue-specific dosage disruption

**Fig. 1:**
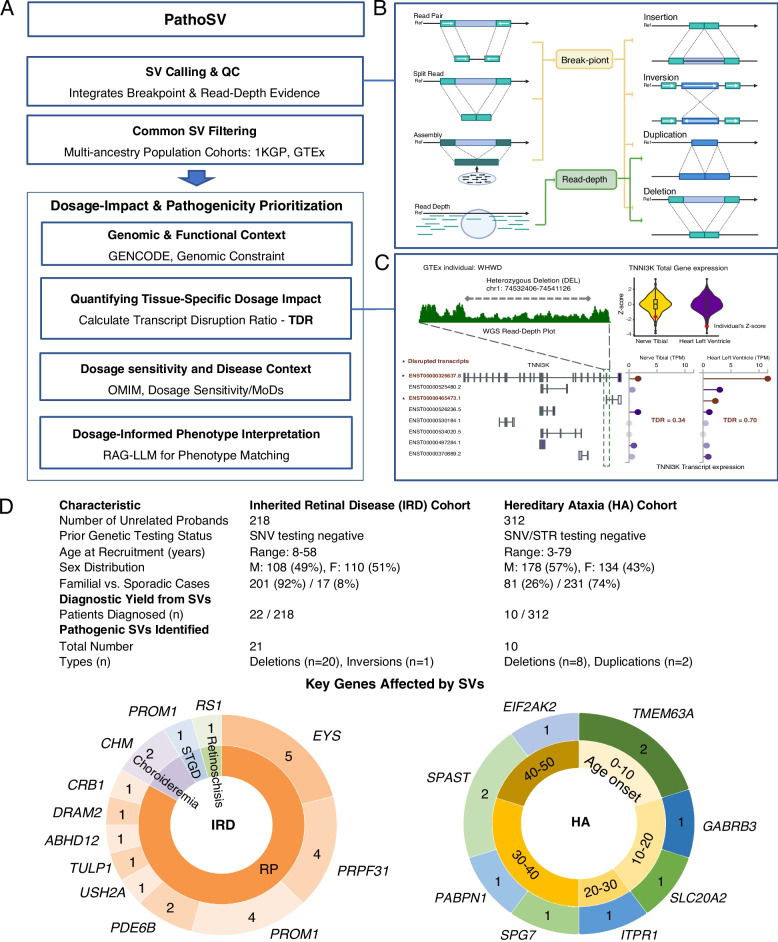
The PathoSV framework for evaluating tissue-specific dosage altering effects of SVs. **A** An overview of the PathoSV workflow. The workflow integrates three main stages: (1) High-confidence SV calling and QC using evidence from both breakpoint and read-depth signals; (2) Common SV filtering against multi-ancestry population cohorts; and (3) Dosage-impact and pathogenicity prioritization. **B** Molecular signatures used for SV detection. Breakpoint evidence (from read pairs, split reads, and assembly) is used to detect duplications, deletions, insertions and inversions. Duplications and deletions are supported by both signal types. **C** An example of characterizing tissue-specific dosage impact with the Transcript Disruption Ratio (TDR). TDR quantifies the functional impact of an SV in a specific tissue. The example demonstrates this principle with a heterozygous deletion in the *TNNI3K* gene from a GTEx individual WHWD. The WGS read-depth plot visually confirms the one-copy loss. Due to different transcript expression patterns between tissues, this deletion results in a TDR of 0.34 in the tibial nerve but a much higher TDR of 0.70 in the heart's left ventricle, leading to a greater downregulation of total gene expression (Z-score) in the heart. **D** Diagnostic summary of dosage disrupting SVs in two neurodegenerative cohorts. The table details the characteristics and diagnostic yield for the Inherited Retinal Disease (IRD) and Hereditary Ataxia (HA) cohorts. The donut charts illustrate the landscape of key genes where pathogenic SVs were identified. In the IRD cohort, pathogenic variants were found in crucial retinal genes such as *EYS* and *PRPF31*. In the HA cohort, the chart displays causative genes like *SPAST* and *ITPR1*, organized by the patient's age of onset

To systematically evaluate the tissue-specific dosage disrupting effects of SVs in the human genome, we developed a computational framework (PathoSV) designed to characterize an SV's disruptive impact on gene dosage within a disease-relevant tissue (Fig. 1A). We first employed an SV detection strategy that combines evidence from methods sensitive to different molecular signatures of SV formation (Fig. 1B). We use breakpoint calls [31] as the primary input and leverage corroborating evidence from read-depth analysis [32, 34] for quality control (Additional file 1: Fig. S1). This integrated approach is intended to reduce the false-positive rates of individual callers.

Leveraging large-scale transcriptomic data, we systematically evaluated the functional impact of rare SVs in the context of specific tissues to quantify their effect on gene dosage. We found that the dosage effect is a characteristic feature of rare SVs, and this effect is tissue specific. Characterizing their transcriptomic profiles, we further derived that Transcript Disruption Ratio (TDR, see Methods) can effectively capture such effects. TDR is a metric that calculates the proportion of a gene's total expression in a specific tissue that is comprised of transcripts directly disrupted by an SV. As illustrated in Fig. 1C, a single deletion can have a markedly different impact across tissues; in the *TNNI3K* gene, the same deletion results in a TDR of 0.34 in the nerve but a higher TDR of 0.70 in the heart, reflecting the different expression patterns of the affected transcripts in each tissue.

We further evaluated tissue-specific dosage disrupting effects of SVs in two large, previously unresolved neurodegenerative disease cohorts, with key results summarized in Fig. 1D. In a cohort of 218 individuals with inherited retinal diseases (IRDs) where prior panel sequencing was inconclusive, PathoSV identified 21 pathogenic SVs (primarily deletions), providing a molecular diagnosis for 22 patients. These variants affected critical retinal genes such as *EYS* and *PRPF31*, which are linked to diseases like Retinitis Pigmentosa (RP). Similarly, in a cohort of 312 patients with hereditary ataxia (HA) who were negative after standard SNV and short tandem repeat (STR) testing, we provided a diagnosis for 10 individuals. We identified 10 pathogenic deletions and duplications in key ataxia-associated genes, including *SPAST* and *ITPR1*. These findings reveal that tissue specific dosage disruption is a rare but distinct pathogenic effect of SVs in the human genome.

### Rare structural variants disrupt gene dosage in a tissue-specific context

**Fig. 2:**
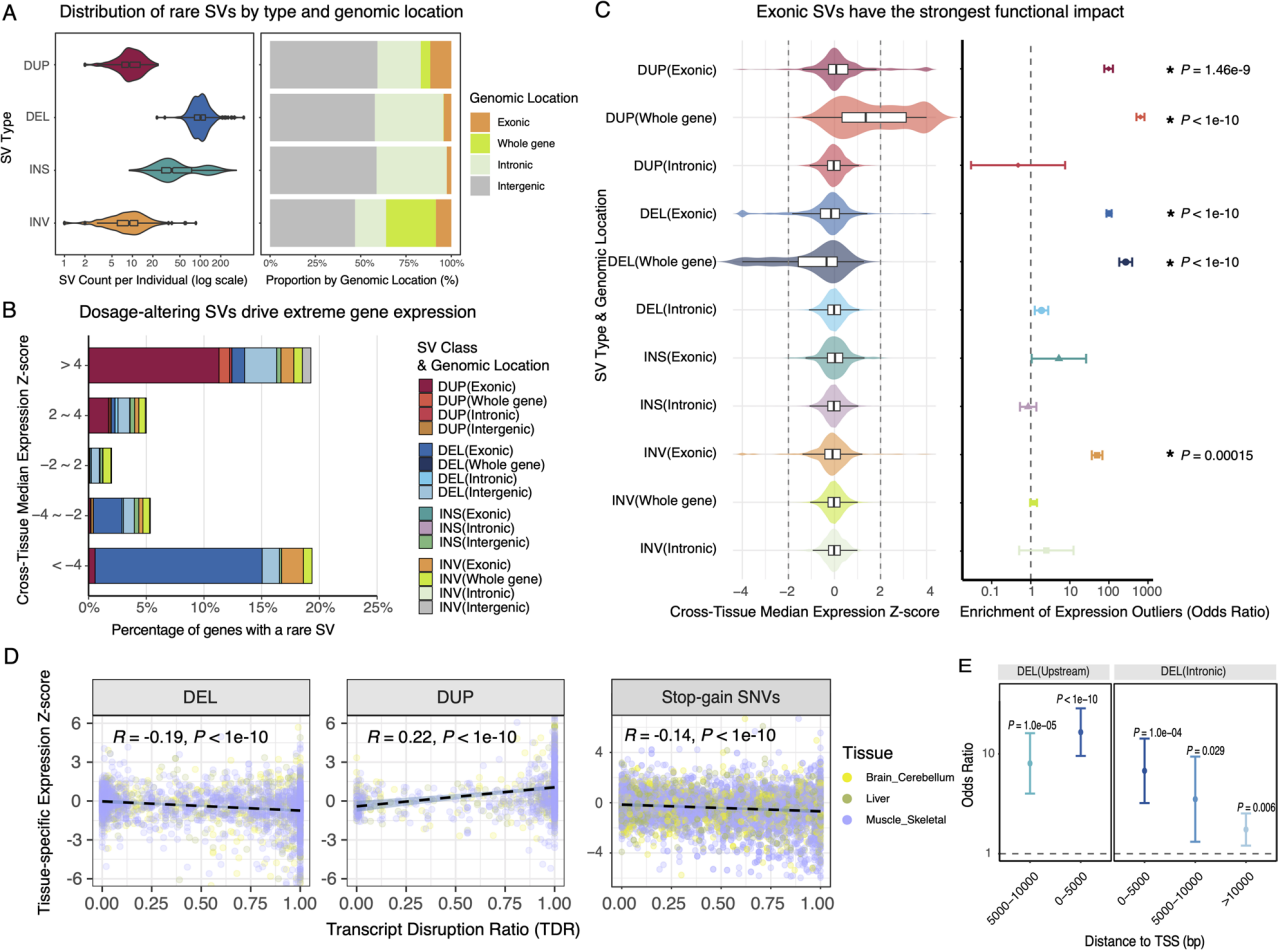
Dosage altering effect and genomic distribution of rare structural variants. **A** Baseline distribution of rare SVs per individual. The violin plot shows the count of different SV types per individual, while the bar chart shows that most SVs reside in non-coding (intronic and intergenic) regions. **B** Enrichment of rare SVs in genes that are expression outliers. The stacked bar chart shows the percentage of genes within different expression bins (cross-tissue median Z-score) that have an associated rare SV, categorized by type and genomic location. Cross-tissue median Z-score summarizes a gene's overall expression level across 49 GTEx tissues to capture systemic, body-wide effects. A strong correlation is evident: extreme under-expression (Z-score < −2) is associated with exonic/whole-gene deletions, while extreme over-expression (Z-score > 2) is associated with exonic/whole-gene duplications. **C** Characterization of SV functional impact. The left panel plots the cross-tissue median gene expression Z-score for different SV types, illustrating their strong directional impact. Exonic and whole-gene deletions are associated with under-expression (negative Z-score), while duplications are associated with over-expression (positive Z-score). The right panel shows the odds ratio for an SV carrier to be a gene expression outlier (defined as |Z-score|> 2), confirming that exonic/whole-gene deletions, duplications, and exonic inversions are enriched for driving extreme expression changes. Error bars indicate the 95% confidence interval, and asterisks denote statistical significance (*P* < 0.05). **D** The Transcript Disruption Ratio (TDR) quantitatively correlates with the magnitude of gene dosage alteration in a tissue-specific manner. Scatter plots show the relationship between the TDR of rare SVs and the tissue-specific gene expression Z-score for the affected gene in the GTEx cohort. A significant negative correlation is observed for deletions (DEL), where a higher TDR is associated with stronger gene under-expression. Conversely, a significant positive correlation is observed for duplications (DUP), linking higher TDR to stronger over-expression. Correlation coefficient (*R*) and significance (*P*-value) are shown for each SV type. Evaluation of stop-gain SNVs across GTEx tissues also indicates the metric's applicability to nonsense-mediated decay (NMD). Similar to deletions, the scatter plot reveals a negative correlation: variants with high TDR (disrupting the majority of tissue-specific transcripts) drive downregulation. **E** Disruption of cis-regulatory elements underlies expression outliers. The plot shows the enrichment (Odds Ratio) of gene expression outliers among carriers of non-coding deletions, categorized by their distance to the Transcription Start Site (TSS). A significant enrichment is observed for deletions located proximal to the TSS, likely reflecting the disruption of promoter regions. This highlights that disruption of cis-regulatory elements is also a key factor underlying expression anomalies, independent of coding sequence alteration. Error bars represent 95% confidence intervals. Significance levels are indicated by *P*-values

Leveraging large scale transcriptomic data [2, 17] (838 individuals across 49 tissues), we evaluated the dosage altering effects of SVs. Here we consider rare SVs only (defined as having a population allele frequency < 0.01) as those are most relevant to pathogenicity. Across tissues, there is a strong and a predictable link between rare SVs and gene expression outliers (Fig. 2). As expected from genomic architecture and strong selective pressure against disruptive variants, the vast majority of rare SVs reside in non-coding regions (Fig. 2A). However, while representing only a small fraction of all genomic variants, SVs are a disproportionately large driver of extreme gene dosage changes, with the most potent of these effects caused by SVs that are exonic or encompass a whole gene (Fig. 2B).

We observed a clear directional effect based on SV type and location. Individuals carrying rare deletions that were exonic or encompassed a whole gene were significantly more likely to show extreme under-expression of that gene (Z-score < −2). Conversely, individuals with exonic or whole-gene duplications were enriched for extreme over-expression (Z-score > 2). Considering both directions (|Z-score|> 2), this dosage-disrupting effect was most pronounced for variants directly impacting coding regions, with exonic deletions, duplications, and inversions showing the highest odds of causing a gene expression outlier (Fig. 2C).

Although SVs cause cross-tissue dosage alterations, the effects of these disruptions can vary widely between tissues (Fig. 1C). Due to alternative splicing, a single gene can produce many transcript isoforms with unique expression profiles across different tissues. To characterize this tissue-specific context, we derived the Transcript Disruption Ratio (TDR, see Methods). By correlating TDR with tissue-specific gene expression Z-scores, we found that TDR quantitatively reflects gene dosage alterations, revealing an evident relationship between the transcript-level disruption and the actual dosage impact in the specific tissue (Fig. 2D, Additional file 1: Fig. S3 and Fig. S4). A significant negative correlation was observed for deletions (DEL), where a higher TDR, indicating greater transcript disruption, was strongly associated with more severe gene under-expression. Conversely, a significant positive correlation was evident for duplications (DUP), linking a higher TDR to stronger gene over-expression. TDR is therefore a quantitative predictor of the magnitude of dosage disruption in the relevant tissue context.

Examining the TDR metric beyond SVs, we evaluated stop-gain variants across GTEx tissues. The dosage effect of nonsense mutations is similarly context-dependent, triggering nonsense-mediated decay (NMD) primarily when the variant disrupts transcripts actively expressed in the target tissue. Indeed, we observed a negative correlation between TDR and tissue-specific expression Z-scores (Fig. 2D). This suggests that TDR is a transcriptome-aware metric capable of quantifying dosage impact in a tissue-specific manner across variant classes. While TDR effectively captures dosage changes driven by the physical disruption of transcript structure, it does not currently account for non-coding regulatory effects. By specifically examining deletions in non-coding regions (where TDR = 0), we also observed an enrichment of expression outliers driven by variants located proximal to the transcription start site (Fig. 2E). This enrichment decays rapidly with distance from the TSS, suggesting that the loss of cis-regulatory elements, particularly promoters, is likely a pathogenic mechanism complementing transcript disruption. This highlights regulatory disruption as an important pathogenic mechanism that, although not covered by the current TDR metric, represents a critical complementary direction for functional interpretation.

**Fig. 3:**
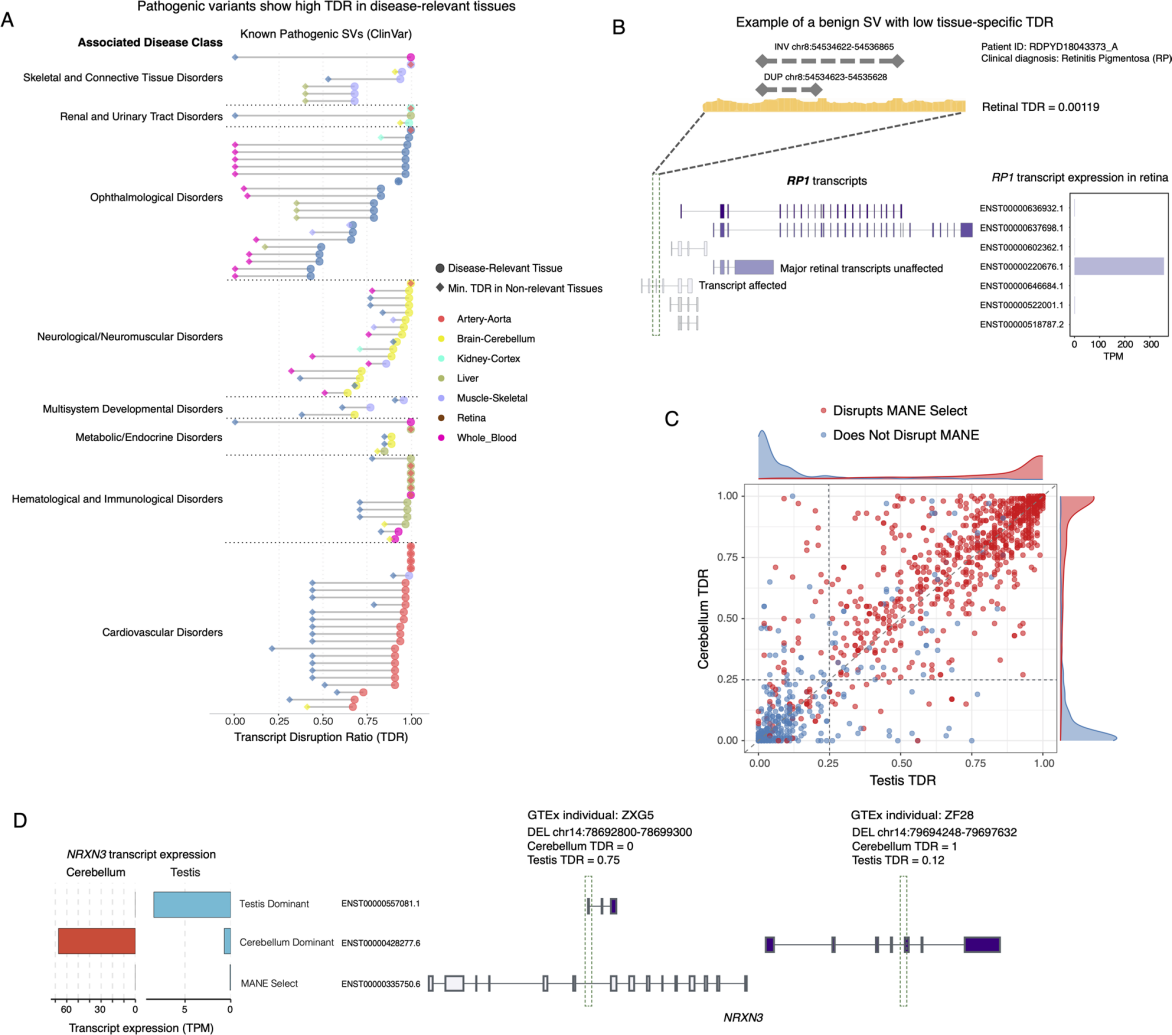
High Transcript Disruption Ratio (TDR) is a key feature of pathogenic variants. **A** Pathogenic SVs exhibit high TDR specifically in disease-relevant tissues. Dumbbell plot comparing the tissue-specific impact of known pathogenic structural variants (from ClinVar) across distinct disease categories. For each variant, the colored circle represents the TDR in the disease-relevant tissue (e.g., Retina for ophthalmological disorders), while the diamond represents the minimum TDR observed across a panel of representative non-disease-relevant tissues (or unaffected). Pathogenic variants consistently show high TDR values (approaching 1.0) in the affected tissue, whereas their impact on other tissues varies widely and is frequently lower, highlighting that dosage disruption is often a tissue-specific event. **B** A contrasting example of benign SVs in a patient with Retinitis Pigmentosa. Although the SV is near the disease gene *RP1*, it has a near-zero retinal TDR (0.00119). This is because the SV only disrupts transcripts that are not expressed in the retina, while the major retinal transcripts are unaffected, explaining the variant's benign nature. These SVs are common polymorphisms and can be found in unaffected family members. **C** Evaluation of SV impact on MANE Select versus non-MANE transcripts. The scatter plot compares the TDR of rare SVs (deletions and duplications) in the Testis (X-axis) versus the Cerebellum (Y-axis). Variants are colored by whether they disrupt the MANE Select transcript (Red) or not (Blue). While SVs disrupting MANE Select transcripts consistently show high TDRs across tissues (diagonal clustering), a subset of non-MANE SVs (~ 12%) exhibits high, tissue-specific TDRs (> 0.25, off-diagonal blue points), highlighting pathogenic effects missed by restricting analysis to MANE transcripts alone. Marginal density plots show the distribution of TDRs for each group. This highlights the transcriptome-awareness of TDR in capturing tissue-specific pathogenicity. **D** Example of tissue-specific isoform switching missed by static annotation. The bar chart displays the expression (TPM) of different *NRXN3* transcript isoforms in the Cerebellum and Testis. Two distinct deletions in *NRXN3* identified in the GTEx dataset demonstrate tissue specific dosage disruption not captured by the MANE Select transcript. The first deletion (left) disrupts a testis-dominant isoform (Testis TDR = 0.75) but spares the cerebellum-dominant isoform (Cerebellum TDR = 0). Conversely, the second deletion (right) disrupts a cerebellum-dominant isoform (Cerebellum TDR = 1.0) while having minimal impact in the testis (Testis TDR = 0.12)

Further evaluation using known pathogenic SVs from the ClinVar [29] database shows that these disease-causing variants consistently have very high TDRs in their respective disease-relevant tissues, with most values approaching 1.0 (Fig. 3A). This indicates that pathogenic SVs cause significant dosage alterations by disrupting the vast majority of a gene's expressed transcripts. In contrast, a benign SV near the disease gene *RP1* in an individual (from the IRD cohort) has a near-zero retinal TDR (0.00119) because it only impacts transcripts that are not expressed in the retina, leaving the major functional isoforms intact (Fig. 3B). These findings suggest that tissue-specific dosage disruption is a key feature of rare SVs while a high dosage disruption effect is a defining feature of pathogenicity.

To quantify the added value of tissue-specific annotation over static canonical references, we compared TDR against the MANE Select transcript set [45]. While the MANE Select transcript set provides a robust, universal reference for clinical interpretation, gene expression is inherently tissue specific. Comparing TDR distributions in distinct tissues (e.g., Cerebellum vs. Testis, Fig. 3C, Additional file 1: Fig. S5), we found that SVs disrupting MANE Select transcripts typically yield high TDRs across tissues (~ 94% with TDR > 0.25), confirming MANE as a highly effective proxy for global high-impact variants. However, reliance on MANE alone overlooks a significant fraction of pathogenic potential. Approximately 12% of SVs that do not disrupt the MANE Select transcript still result in a high TDR (> 0.25) in specific tissues, which arises from tissue-specific isoform usage. For example, in the *NRXN3* gene, the dominant transcript expressed in the cerebellum differs from that in the testis (Fig. 3D), while the MANE Select transcript is not expressed in either tissue. Thus, the transcriptome-aware TDR metric offers the sensitivity to identify tissue-specific pathogenic mechanisms. We also observed that TDR is more discriminative for complex genes: as gene complexity (transcript or exon count) increases, TDR distributions broaden (Additional file 1: Fig. S6). This underscores the metric’s utility to quantify partial transcript disruption and isoform-specific effects.

### Pathogenic dosage-disrupting structural variants are rare in the human genome

**Fig. 4:**
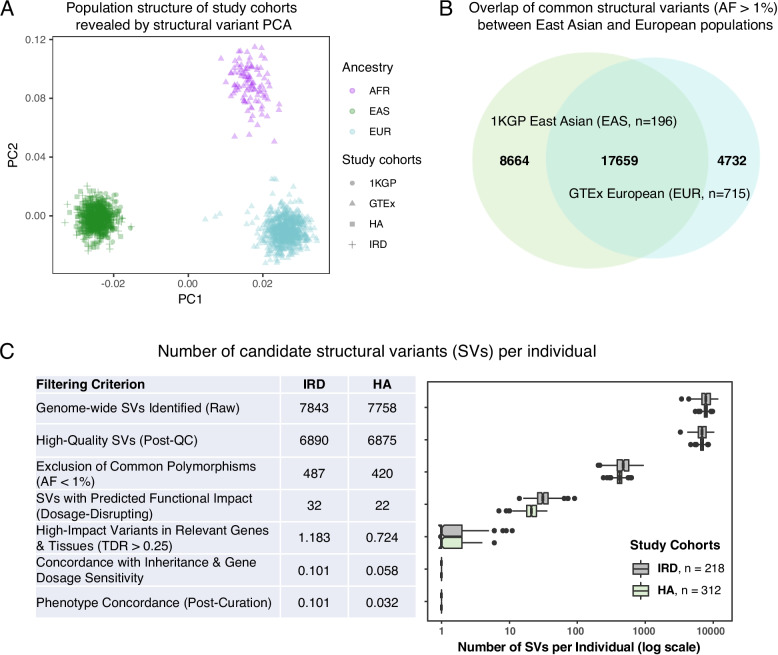
Multi-ancestry filtering reveals that pathogenic dosage-disrupting SVs are extremely rare. **A** Common SVs are shaped by population structure. A principal component analysis (PCA) of common SVs from the study and reference cohorts (1KGP, GTEx, IRD, HA) shows distinct clustering by genetic ancestry. The analysis includes individuals from the Inherited Retinal Disease (IRD; *n* = 218) and Hereditary Ataxia (HA; *n* = 312) patient cohorts, as well as reference individuals from the 1000 Genomes Project (1KGP: EAS; *n* = 196) and the Genotype-Tissue Expression (GTEx: EUR + AFR; *n* = 818) project. The plot shows clear clustering by continental ancestry, with PC1 primarily separating the East Asian (EAS) group from the African (AFR; *n* = 103) and European (EUR; *n* = 715) groups, while PC2 distinguishes the African group. This highlights that SV allele frequencies are often population specific. **B** Overlap of common SVs between populations. The Venn diagram shows that many common SVs (AF > 1%) are unique to either the East Asian (1KGP) or European (GTEx) reference cohorts, demonstrating the necessity of a multi-ancestry panel to effectively filter benign, population-specific polymorphisms. **C** A multi-tiered filtering strategy, including removal of common polymorphisms and prioritization of variants with high TDR (> 0.25) in relevant tissues, effectively isolates candidate pathogenic SVs. The table and adjacent box plot illustrate the progressive reduction in the median number of candidate SVs per individual at each filtering step. The steps include: (1) initial raw SV calls; (2) quality control; (3) removal of common variants found in the background cohorts; (4) prioritization of variants predicted to be dosage-disrupting (i.e., exonic/whole-gene deletions and duplications, or exonic inversions); (5) filtering for variants with a high TDR (> 0.25) in the relevant tissue of disease-associated genes; (6) assessment for segregation consistent with the expected inheritance pattern or dosage sensitivity; and (7) final manual curation to identify pathogenic candidates. This cascade effectively reduces the number of candidate SVs from a median of ~ 7,000 per individual to fewer than 1/10, isolating the most likely causative variants for each patient

We further investigated the abundance of those dosage altering SVs and those tissue-specific effects that are likely to be pathogenic in the average human genome. Although common SVs may also cause gene dosage change [4], their effects are most likely benign. The human genome contains thousands of structural variants, the vast majority of which are common and benign polymorphisms whose frequencies vary across ancestries. The frequencies of many SVs differ significantly between populations due to genetic drift. A principal component analysis (PCA) based on common SVs shows clear clustering of individuals by their continental ancestry (Fig. 4A). To identify pathogenic dosage effects therefore also requires the exclusion of those common SVs. However, a variant filtering strategy based on a single-ancestry reference panel would incorrectly retain many population-specific common variants. For example, thousands of common SVs (AF > 1%) are unique to either East Asian or European populations (Fig. 4B). To characterize such population structures, we built a comprehensive, multi-ancestry background database from 1,014 individuals of East Asian (*n* = 196), European (*n* = 715), and African (*n* = 103) ancestry. To create this harmonized resource, we re-processed all raw sequencing reads from these public cohorts using the same bioinformatic pipeline applied to our patient samples. By removing any SV with an allele frequency (AF) > 1% in this combined cohort, we effectively eliminated population-specific polymorphisms and substantially enriched our dataset for truly rare variants.

To investigate the abundance of dosage changing SVs and those that are likely to be pathogenic, we employed a multi-tiered filtering cascade. After stringent quality control, the number of high-quality SVs per genome is ~ 6,800. The multi-ancestry allele frequency filter substantially cuts this number to ~ 450 rare variants per person. Among these, cross-tissue dosage-disrupting SVs, such as exonic deletions or duplications, are typically in the range of 20–30 per individual. Applying the tissue-specific TDR filter (TDR > 0.25) in disease-relevant genes, leaves, on average, less than one candidate SV per individual (Fig. 4C). These results demonstrate that candidate pathogenic SVs causing tissue-specific dosage alterations in disease-relevant tissues are extremely rare among populations, consistent with strong selective pressure against such variants.

### Dosage-disrupting SVs underlie a significant fraction of unresolved inherited neurodegenerative disease

To evaluate their pathogenic effects, we further investigated tissue-specific dosage disruption effects of SVs in two cohorts of patients with neurodegenerative disorders who remained undiagnosed after standard genetic testing. In a cohort of 218 unrelated patients with various IRDs, we identified 21 pathogenic SVs (Additional file 1: Fig. S7, Table S2 and Table S3), providing a molecular diagnosis for 22 individuals (10.1% diagnostic yield). The identified variants were predominantly large deletions affecting genes critical for retinal function, such as *EYS* and *PRPF31*, which are established causes of Retinitis Pigmentosa (RP). For these autosomal dominant genes like *PRPF31*, a 50% reduction in gene dosage is a known pathogenic mechanism [46], consistent with our findings of whole-gene or multi-exon deletions that result in haploinsufficiency.

**Fig. 5:**
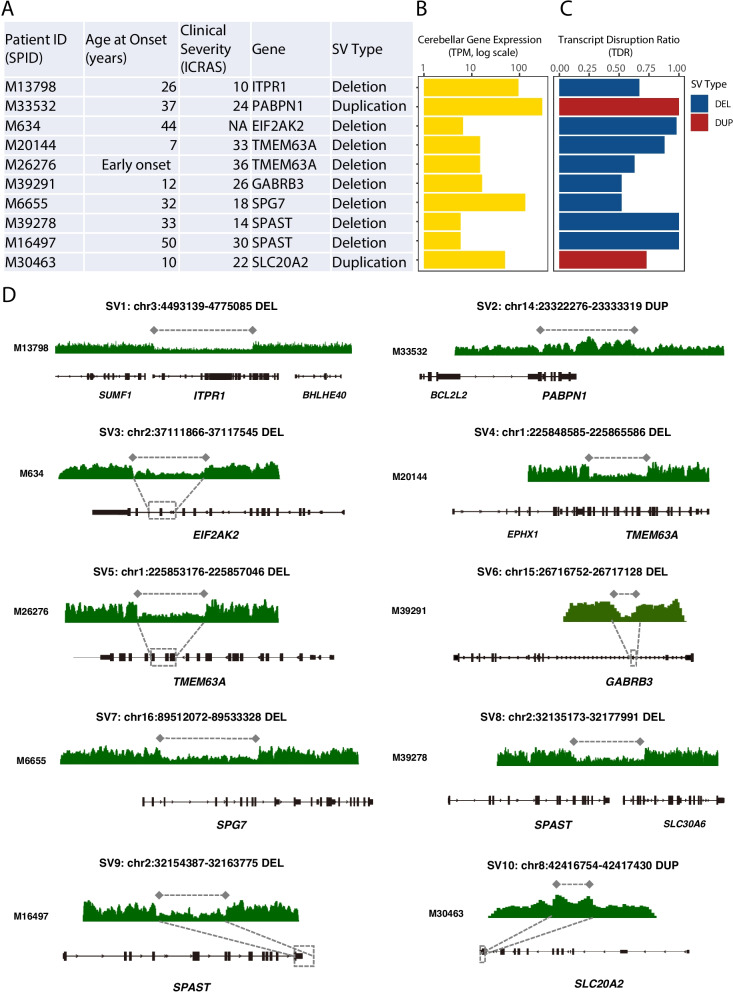
Pathogenic dosage disrupting structural variants identified in the Hereditary Ataxia (HA) cohort. **A** A summary of clinical and genetic details for 10 individuals with a molecular diagnosis. Patient ID (SPID), age at onset, clinical severity as measured by the International Cooperative Ataxia Rating Scale (ICARS), the affected gene, structural variant (SV) type, and its functional consequence are listed. **B** Expression levels of the affected genes in the cerebellum. Bars represent the median transcripts per million (TPM) for each gene from the GTEx cerebellum reference dataset. This demonstrates that all affected genes are expressed in the relevant neural tissue. **C** The functional impact of each SV as quantified by the Transcript Disruption Ratio (TDR). The TDR calculates the proportion of cerebellum-specific transcripts that are disrupted by the SV. A higher TDR indicates a greater impact on the gene's functional output. All candidate pathogenic SVs have a high TDR (> 0.25), consistent with a significant functional consequence. **D** Pathogenic structural variants identified in the HA cohort. The figure displays the 10 pathogenic structural variants (SVs) that provided a molecular diagnosis for 10 patients in the HA cohort. These variants consist of eight deletions and two duplications. Each panel (SV1-SV10) shows a unique SV, with a read-depth plot (green tracks) illustrating the copy number change over the affected gene locus. A visible decrease in sequencing coverage confirms a deletion, while an increase indicates a duplication. The identified SVs impact key ataxia-associated genes, including *ITPR1*, *PABPN1*, *EIF2AK2*, *TMEM63A*, *GABRB3*, *SPG7*, *SPAST*, and *SLC20A2*. All variants were prioritized for their high predicted functional impact in the cerebellum, reflected by a TDR greater than 0.25

In a cohort of 312 unrelated patients with HA who were negative for prior SNV/STR testing, we identified 10 pathogenic SVs, providing a molecular diagnosis for 3.2% of previously unresolved cases (10/312) (Fig. 5, Additional file 1: Table S4 and Table S5). These variants included eight deletions and two duplications affecting key cerebellar function genes, all of which were expressed in the cerebellum and had high cerebellar TDRs (> 0.50), indicating a significant impact on gene dosage (Fig. 5B, C). The identified SVs correlated with the patients' clinical presentations. For instance, deletions in *SPAST* and *SPG7* were found in patients with spastic gait and hyperreflexia, classic features of Hereditary Spastic Paraplegia (HSP) [47]. These genes encode microtubule-severing and mitochondrial proteins, respectively, and are known to be dosage-sensitive; a loss of one copy is sufficient to impair neuronal function. Similarly, a deletion in *ITPR1*, a critical calcium channel, was identified in a patient with spinocerebellar ataxia (SCA15) [48], a disorder caused by impaired cerebellar calcium signaling. These findings demonstrate that the pathogenic SVs of tissue-specific dosage disruption effect represent a rare but distinct pathogenic mechanism.

## Discussion

In this study, we investigated structural variants in a tissue-relevant transcriptomic context. The effect of an SV is a consequence of its interaction with the unique transcriptomic landscape of the affected tissue. We have previously observed strong cross-tissue dosage altering effect of SVs [2, 4], however such effects do not account for the functional complexities arising from alternative splicing and tissue-specific expression [8], as an SV's functional consequence depends on which specific transcripts it disrupts within the relevant tissue context.

We conducted a systematic investigation of such tissue-specific effects, leveraging large scale transcriptomic data, and found that tissue-specific dosage disruption is a major property of rare SVs. Such tissue-specific effects are widespread in the human genome and can be characterized by the Transcript Disruption Ratio (TDR), which is the proportion of a gene's functional output (measured by transcript expression) compromised by an SV in a specific tissue. TDR serves as an effective indicator of dosage disruption, providing a tissue-specific metric of the impact of SVs on gene expression. Analysis of known pathogenic variants from ClinVar showed that a high TDR in the disease-relevant tissue is a defining feature of true disease-causing SVs.

Evaluating tissue specific dosage altering effects of SVs also reveals their pathogenic effect. The tissue-specific effect of structural variants (SVs) is particularly important for neurodegenerative conditions like inherited retinal diseases (IRDs) and hereditary ataxias (HAs), where dysfunction in neural tissues with highly specialized transcriptomic features drives pathology [11, 12]. Our prior work observed that dosage sensitivity is an intrinsic, tissue-specific property of genes, with the nervous system being particularly enriched for dosage-sensitive genes [15]. Evaluating tissue specific dosage altering effects of SVs yielded new diagnoses in previously unresolved patient cohorts. In previously unresolved IRD cases, SVs generate a diagnostic yield of 10.1%, while in SNV/STR-negative HA patients, SVs provided a diagnosis for 3.2% of cases. The identified pathogenic SVs in both cohorts resided in genes known to be highly expressed and functionally important in the retina (e.g., *EYS*, *PRPF31*) and cerebellum (e.g., *ITPR1*, *SPG7*), respectively, and all exhibited high TDR values. The nervous system's particular vulnerability to dosage disruption, as suggested by our findings, likely stems from the reliance on non-redundant systems such as signal amplification cascades sensitive to key hubs (e.g., *ITPR1*), metabolic networks dependent on efficient axonal transport (e.g., *SPAST*), and the pressure to maintain structural proteostasis (e.g., *EYS*). Our findings support tissue-specific dosage disruption, as quantified by TDR, as an important determinant of SV pathogenicity.

To further advance SV interpretation, integrating single-cell transcriptomics [49] would be an important next step, enabling the resolution of cell-type-specific dosage disruption that is often obscured in bulk tissue profiles. Concurrently, the broader adoption of stranded long-read sequencing [50] should help refine isoform-specific quantifications, resolving the ambiguities currently inherent in short-read RNA-seq. Finally, extending these frameworks to encompass non-coding regulatory perturbations will also be important for building a more comprehensive view of SV pathogenicity.

## Conclusions

We show that tissue-specific dosage disruption is a key feature of rare SVs in the human genome, which is consequently an important pathogenic mechanism in genetic disorders. Given the broad relevance of gene dosage sensitivity to disease, tissue-specific dosage disruption induced by SVs may contribute to a proportion of currently unresolved Mendelian disorders.

## Supplementary Information

Additional file 1: Supplementary figures and tables. Fig. S1, SV filtering workflow; Fig. S2, short-read/long-read isoform concordance; Fig. S3, deletion TDR–expression correlations across tissues; Fig. S4, duplication TDR–expression correlations across tissues; Fig. S5, TDR versus MANE comparison; Fig. S6, TDR and gene structural complexity; Fig. S7, IRD pedigrees and genomic evidence. Table S1, bioinformatics pipeline and resources; Table S2, IRD pathogenic variant molecular data; Table S3, IRD clinical phenotypes; Table S4, HA structural variant molecular data; Table S5, HA clinical phenotypes.

## Data Availability

The PathoSV source code, analysis scripts, background SV frequency database, and reference files used in this study are available at GitHub: https://github.com/xlilab/PathoSV [37]. A web interface for PathoSV, which incorporates an LLM using retrieval-augmented generation (RAG) for interpretation support, is accessible at https://www.biosino.org/pathosv/ [41]. The Map of Dosage sensitivity (MoDs) metric and code are available at GitHub: https://github.com/xlilab/MoDs [15]. GTEx v8 RNA-seq and whole-genome sequencing data are available through dbGaP under accession phs000424.v8.p2 [17], and transcript expression data are available through the GTEx Portal: https://gtexportal.org/home/datasets [18]. 1000 Genomes Project data are available at https://www.internationalgenome.org/data [16]. Clinical phenotypes and the molecular characteristics of all pathogenic variants identified in the diagnosed IRD and HA cases are provided in Additional file 1: Tables S2-S5. Processed genomic data for the patient cohorts, including VCF files of called structural variants and de-identified sample information for the Inherited Retinal Disease (IRD) and Hereditary Ataxia (HA) cohorts, have been deposited in the Genome Variation Map (GVM), National Genomics Data Center, accession number GVM001353, within BioProject PRJCA060409 [51]. Access to these data is subject to approval by the Data Access Committee in accordance with participant consent and the regulations of the Ministry of Science and Technology of China regarding Human Genetic Resources. Qualified researchers may apply to the Data Access Committee (DAC) by submitting a Data Access Request (DAR) at https://ngdc.cncb.ac.cn/bioproject/browse/PRJCA060409. Applicants will be required to provide a description of the proposed research project and data security protocols, and sign a Data Use Agreement (DUA) which restricts data use to biomedical research purposes only, prohibits any attempt to re-identify participants, and prohibits the redistribution of data.

## Abbreviations

GTEx: Genotype-Tissue Expression Project
SV: Structural variant
TDR: Transcript Disruption Ratio
CNV: Copy number variant
DEL: Deletion
DUP: Duplication
INS: Insertion
INV: Inversion
1KGP: 1000 Genomes Project
AF: Allele frequency
HA: Hereditary Ataxia
IRD: Inherited Retinal Disease
LoF: Loss-of-function
MANE: Matched Annotation from NCBI and EMBL-EBI
MoDs: Map of Dosage sensitivity
OMIM: Online Mendelian Inheritance in Man
PCA: Principal component analysis
QC: Quality control
SNV: Single nucleotide variant
STR: Short tandem repeat
TPM: Transcripts per million
VCF: Variant call format
WGS: Whole genome sequencing
