# Supplementary material for: Tissue-specific gene dosage disruption is a key feature and pathogenic mechanism of structural variants in the human genome
Source: Genome Med. 2026 Apr 18;18:76. doi: 10.1186/s13073-026-01653-7 (PMC13224669; doi:10.1186/s13073-026-01653-7)
Supplement: Supplementary file 1 — Additional file 1: Supplementary figures and tables. Fig. S1, SV filtering workflow; Fig. S2, short-read/long-read isoform concordance; Fig. S3, deletion TDR–expression correlations across tissues; Fig. S4, duplication TDR–expression correlations across tissues; Fig. S5, TDR versus MANE comparison; Fig. S6, TDR and gene structural complexity; Fig. S7, IRD pedigrees and genomic evidence. Table S1, bioinformatics pipeline and resources; Table S2, IRD pathogenic variant molecular data; Table S3, IRD clinical phenotypes; Table S4, HA structural variant molecular data; Table S5, HA clinical phenotypes. [file 13073_2026_1653_MOESM1_ESM.pdf]

Supplementary Information for:

Tissue-specific gene dosage disruption is a key feature and pathogenic mechanism of structural variants in the human genome

Xubing Liu†, Zhao Chen†, Qian Jiang†, Haoyu Shen†, Zhenguo Wang, Zhe Li, Hongyu Liu, Jihong Wu*, Hong Jiang*, Xin Li*

†These authors contributed equally to this work.
*Correspondence: Xin Li (lixin@sinh.ac.cn); Hong Jiang (jianghong73868@126.com); Jihong Wu (jihongwu@fudan.edu.cn)

Contents

Fig. S1. SV filtering workflow
Fig. S2. Short-read/long-read isoform concordance
Fig. S3. Deletion TDR–expression correlations across tissues
Fig. S4. Duplication TDR–expression correlations across tissues
Fig. S5. TDR versus MANE comparison
Fig. S6. TDR and gene structural complexity
Fig. S7. IRD pedigrees and genomic evidence
Table S1. Bioinformatics pipeline and resources
Table S2. IRD pathogenic variant molecular data
Table S3. IRD clinical phenotypes
Table S4. HA structural variant molecular data
Table S5. HA clinical phenotypes

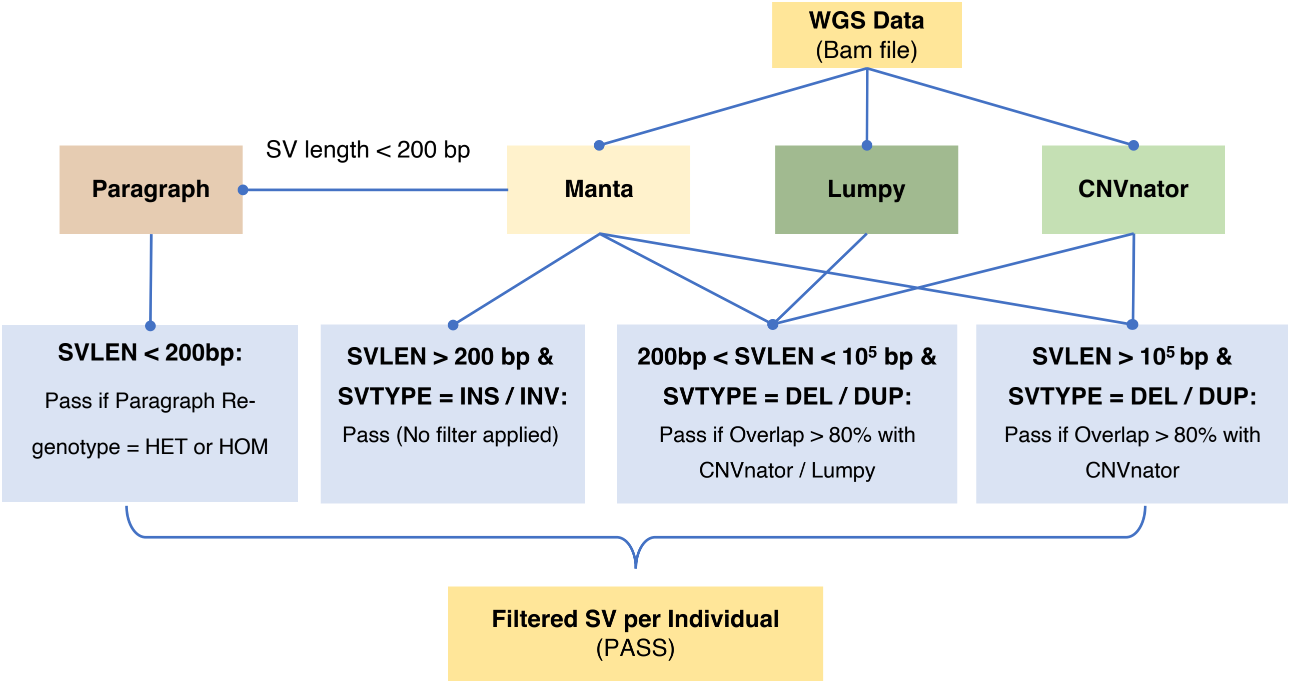

Figure S1. Schematic of the structural variant (SV) filtering workflow. The flowchart details the integrated approach used to enhance the specificity of SV calls from whole-genome sequencing (WGS) data. Initial SV candidates are generated by the primary caller, Manta. These calls are then subjected to a rigorous, size- and type-dependent filtering process that requires corroborating evidence from auxiliary tools. Short SVs (< 200 bp) must be confirmed by a confident genotype (heterozygous, HET; or homozygous, HOM) from the re-genotyper, Paragraph. Medium-sized copy number variants (CNVs)—deletions (DEL) and duplications (DUP) between 200 bp and 100 kb—require > 80% reciprocal overlap with a call from either Lumpy or CNVnator. Large CNVs (≥ 100 kb) require support from the read-depth-based caller, CNVnator. Long insertions (INS) and inversions (INV) do not undergo this overlap-based filtering due to challenges in reliable validation. This workflow yields a high-confidence ('PASS') set of filtered SVs for each individual that is used for downstream analysis.

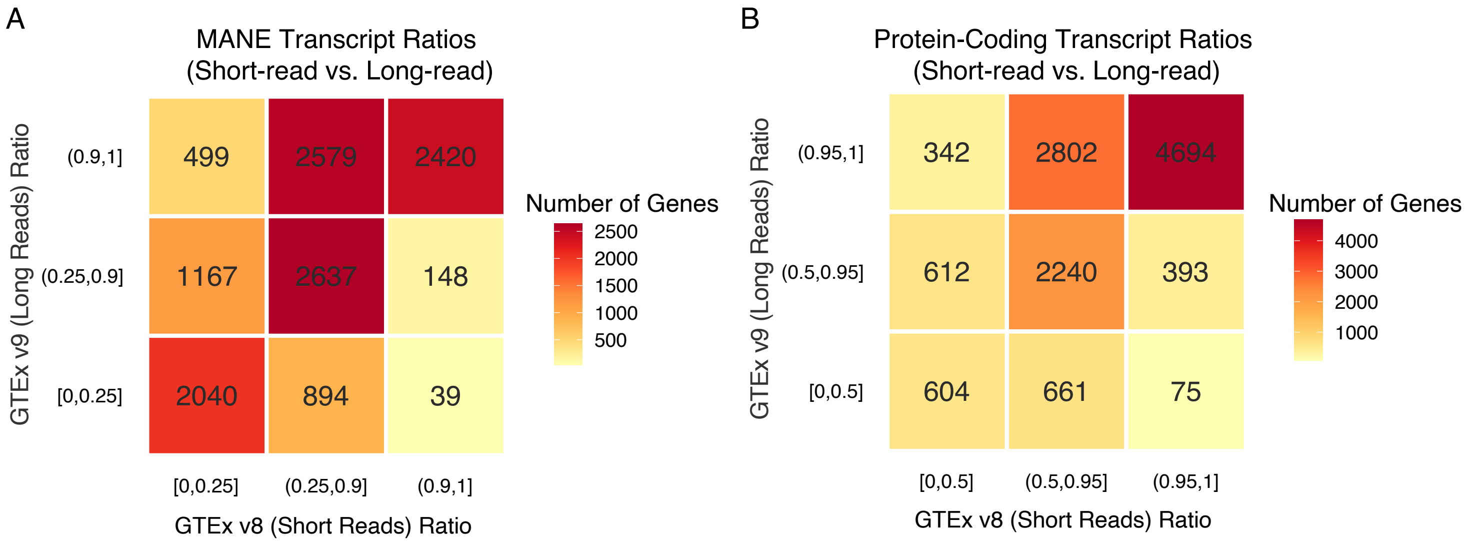

Figure S2. Concordance of transcript isoform ratios between GTEx v8 short-read and v9 long-read data in the frontal cortex. Heatmaps displaying the distribution of transcript ratio counts. The x-axis represents data from GTEx v8 (unstranded short reads), and the y-axis represents data from GTEx v9 (long reads) in frontal cortex samples. GTEx v9 long-read data were generated using Oxford Nanopore Technologies (ONT) via a PCR-cDNA protocol, yielding reads with a median aligned length of 789 bp, providing a high-confidence reference for isoform quantification. **(A)** Comparison of the ratio of the MANE (Matched Annotation from NCBI and EMBL-EBI) transcript expression to total gene expression. **(B)** Comparison of the ratio of total protein-coding transcript expression to total gene expression. Diagonal shading in both panels indicates relatively high concordance between short-read and long-read quantification methods, validating the utility of short-read data for general ratio estimation.

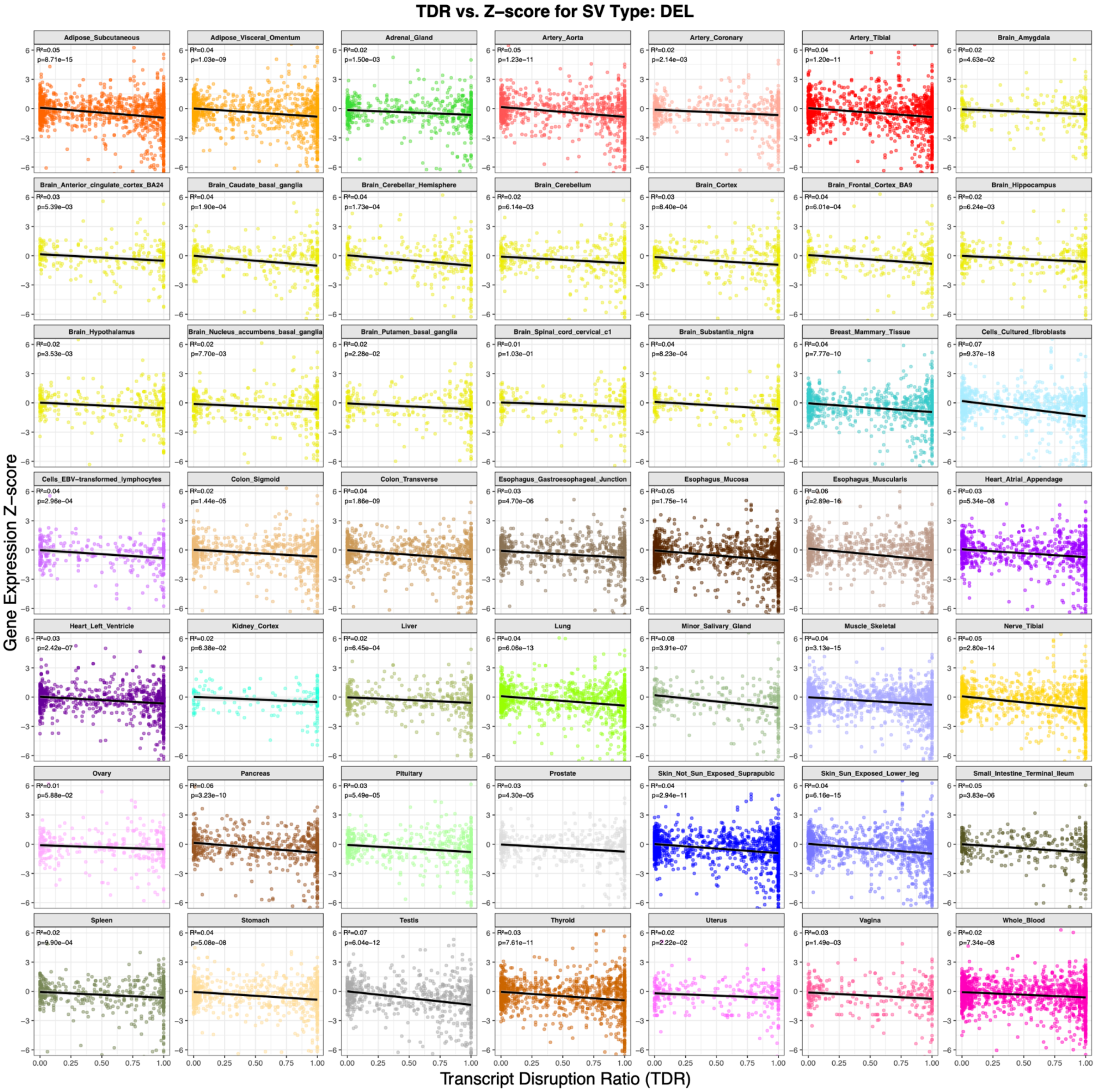

Figure S3. Transcript Disruption Ratio (TDR) versus gene expression Z-score for deletions (DEL) across GTEx tissues. Each panel represents a specific tissue, with the TDR indicating the extent of transcript disruption and the Z-score reflecting gene expression changes.

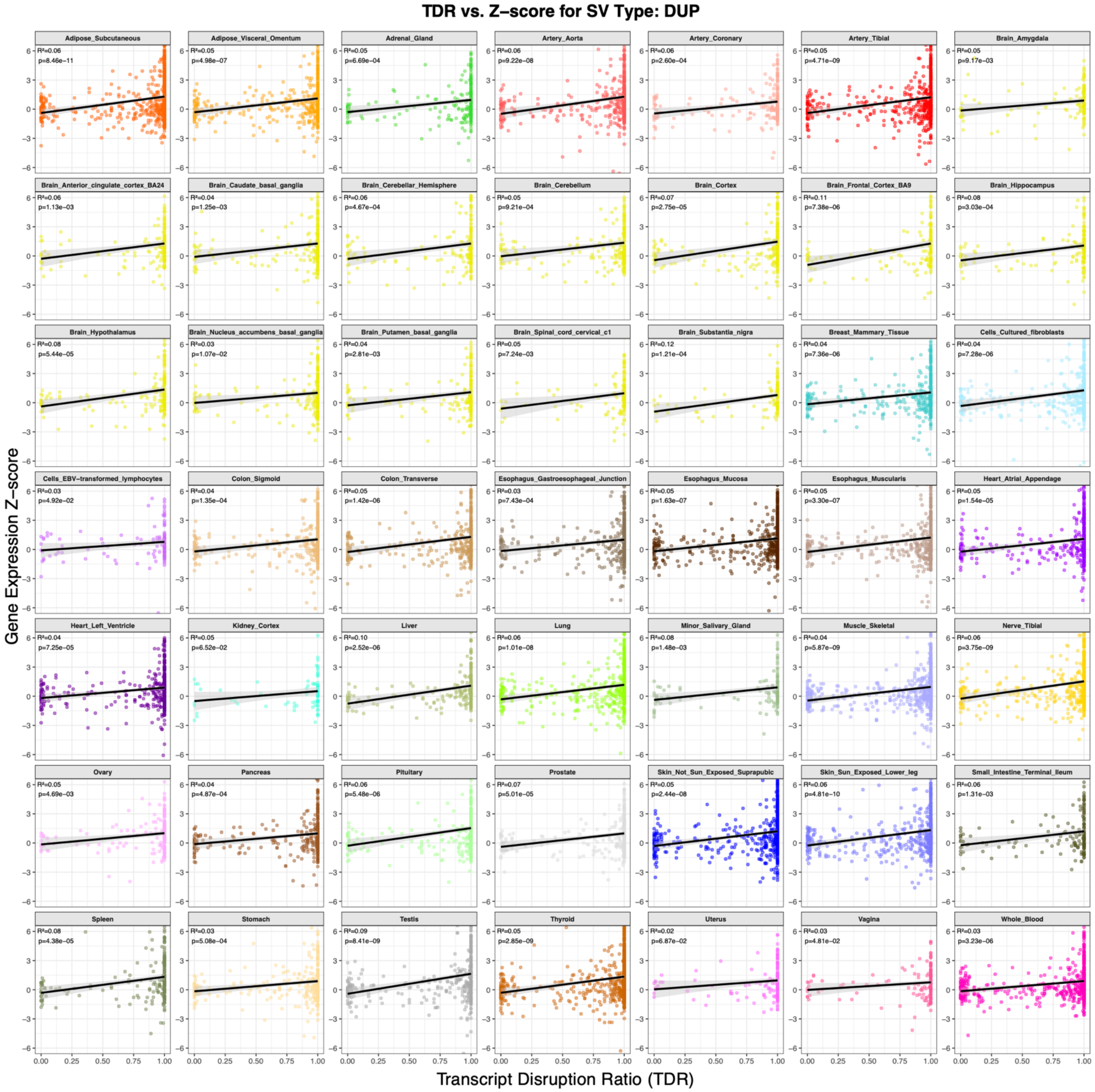

Figure S4. Transcript Disruption Ratio (TDR) versus gene expression Z-score for duplications (DUP) across GTEx tissues. Each panel represents a distinct tissue, with the TDR quantifying the degree of transcript disruption and the Z-score showing the corresponding gene expression alteration.

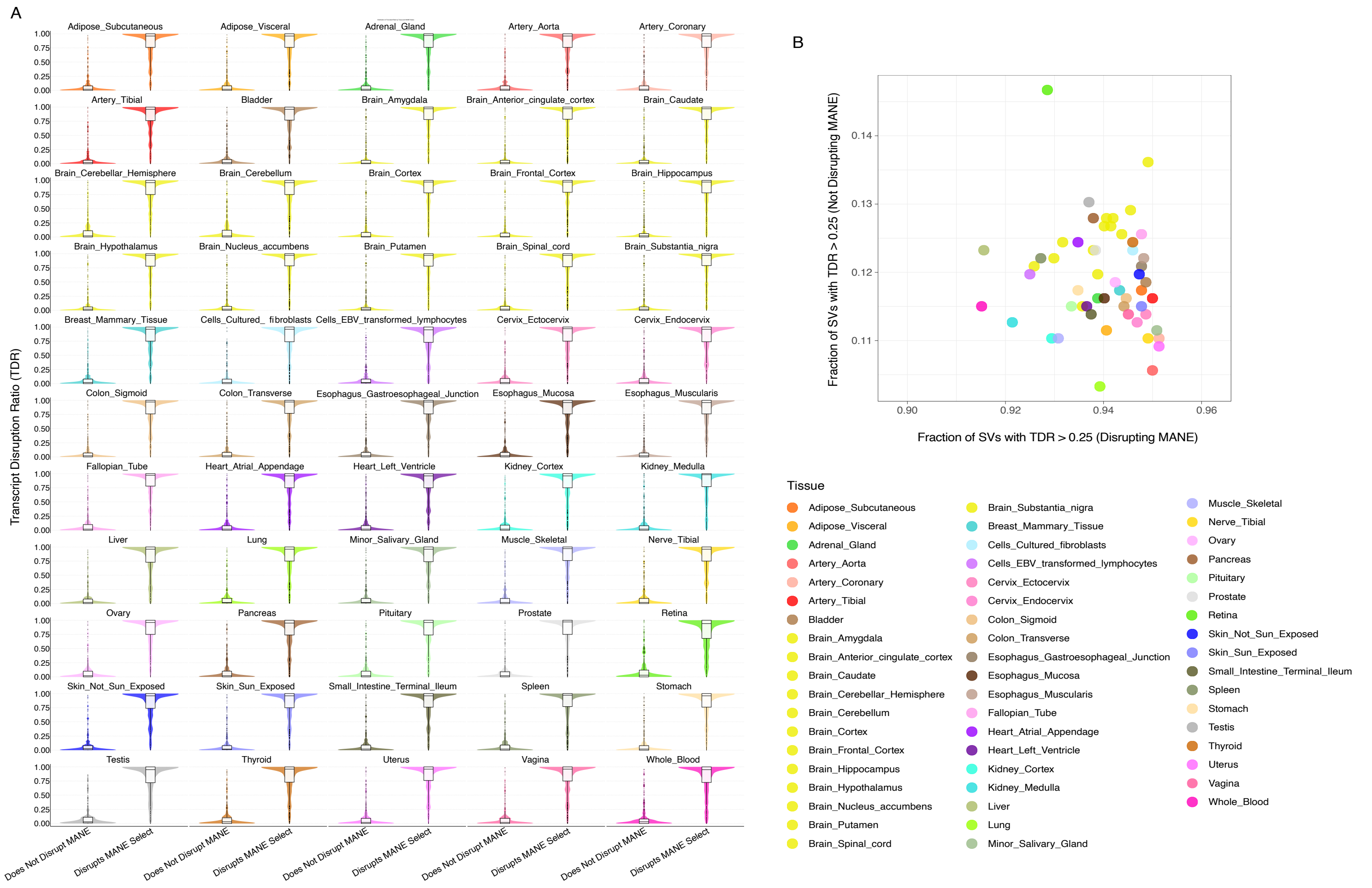

Figure S5. TDR captures tissue-specific dosage disruption beyond static canonical references. (A) Distribution of Transcript Disruption Ratios (TDR) for SVs (deletions and duplications) categorized by their impact on MANE Select transcripts across 49 GTEx tissues. SVs disrupting MANE Select transcripts consistently exhibit high TDRs (approaching 1.0), confirming MANE as a robust proxy for major isoform disruption. However, SVs that do not disrupt MANE transcripts frequently exhibit non-zero TDRs with a long tail extending to high values, indicating potential pathogenicity missed by MANE-restricted annotation. **(B)** Tissue-specific prevalence of high-impact variants among MANE and non-MANE SVs. The scatter plot summarizes the proportion of SVs (deletions and duplications) classified as potentially pathogenic (TDR > 0.25) within each group across tissues. While > 90% of MANE-disrupting SVs are identified as high-impact (X-axis), a significant fraction (~10-15%) of SVs that spare the MANE transcript still result in substantial dosage disruption (TDR > 0.25) in specific tissues (Y-axis). Each point represents a unique tissue type. These findings highlight that while MANE annotations are highly effective, the TDR metric is important for capturing tissue-specific pathogenic events driven by alternative isoform usage.

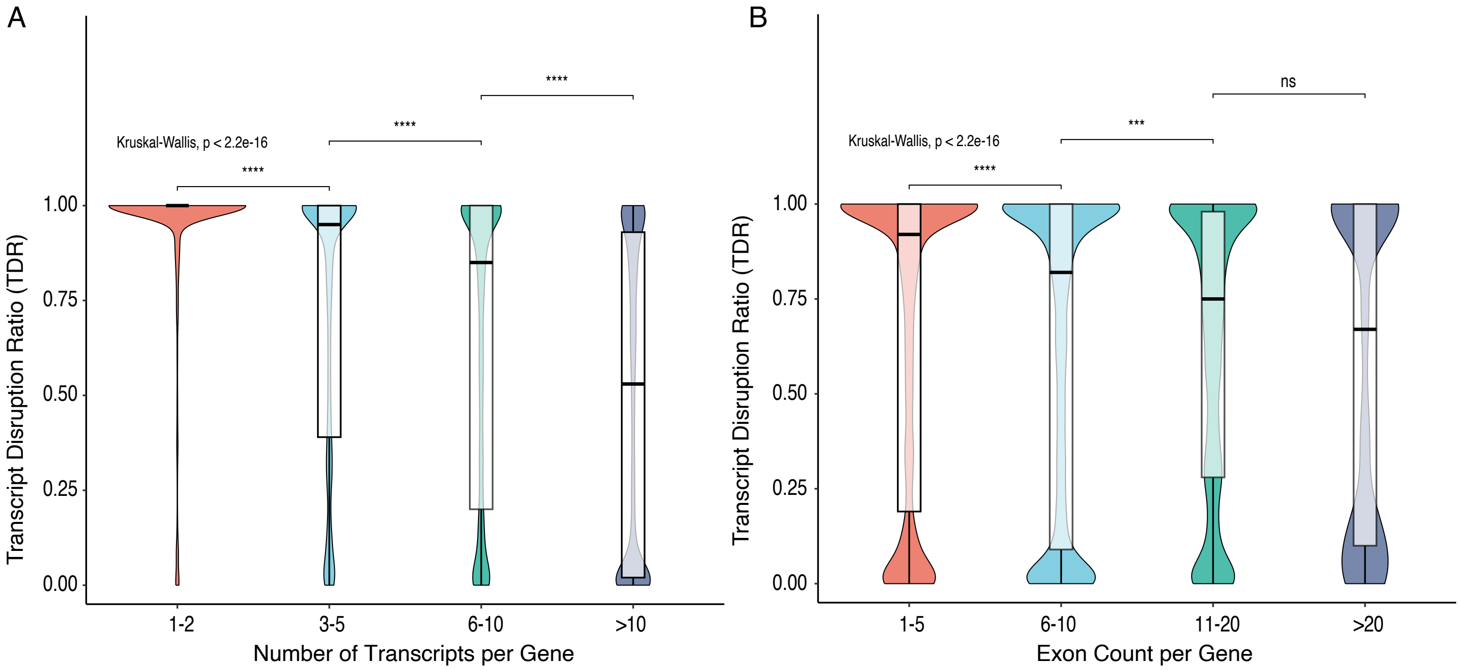

Figure S6. Distribution of Transcript Disruption Ratio (TDR) across genes with varying structural complexity. Violin plots illustrate the distribution of TDR values for SVs (deletions and duplications) identified across GTEx tissues, grouped by gene complexity: transcript counts (left panel) and exon counts (right panel). Increasing gene complexity correlates significantly with a broadening of the TDR distribution and a general shift towards lower median values, contrasting with simple genes (fewer transcripts or exons) which predominantly exhibit clustered TDR values near 1.0. This trend demonstrates the resolution of TDR in capturing partial, likely isoform-specific, disruption events prevalent in complex genes. The internal box plots indicate the median (thick horizontal line) and interquartile ranges. Statistical significance was assessed using the Kruskal-Wallis test with pairwise comparisons indicated.

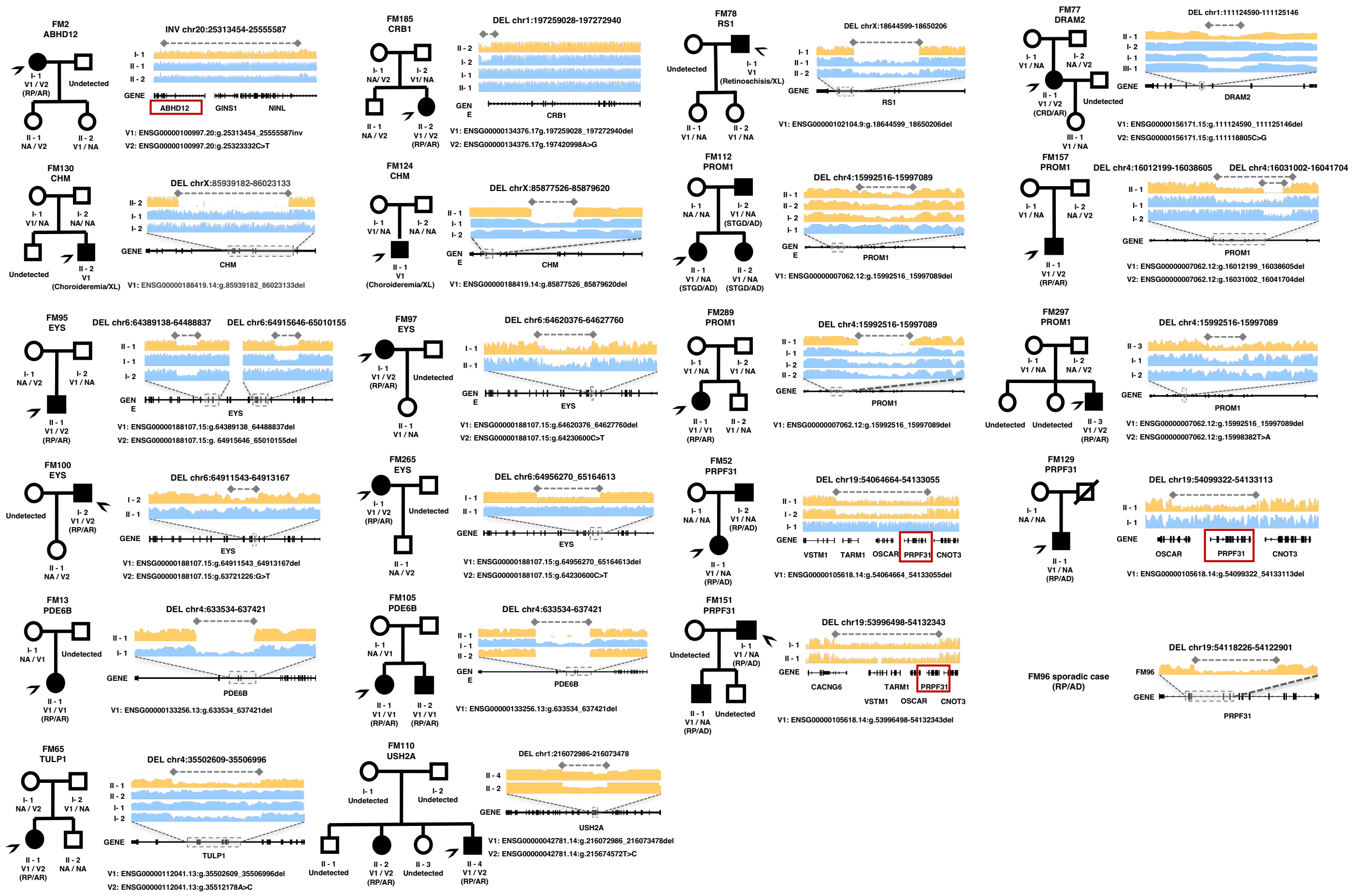

Figure S7. Pathogenic variants identified in 22 families with Inherited Retinal Disease (IRD). The pedigrees and corresponding genomic data for 22 families with a molecular diagnosis of IRD are presented. In each pedigree, circles and squares represent females and males, respectively, while filled shapes indicate affected individuals. A black arrow denotes the proband. Pathogenic variants are labeled as V1, V2, etc., with the wild-type allele designated as "NA" or "Undetected". For a structural variant (SV), an accompanying read-depth plot visualizes sequencing coverage across the affected gene, where a ~50% reduction in coverage indicates a heterozygous deletion.

Table S1: Bioinformatics Pipeline Details and Resources

Category	Tool/Resource	Version	Key Parameters/Notes
Reference Genome	Human Reference Genome	GRCh38/hg38	
Gene Annotation	Gencode	v26	Used for VEP and PathoSV transcript annotation
QC & Alignment	FastQC	v0.11.9	Raw read QC
	BWA-MEM	v0.7.17	Read alignment
	Picard Tools	v2.25.0	MarkDuplicates, CollectInsertSizeMetrics
	GATK	v4.2.6.1	BQSR, HaplotypeCaller, CombineGVCFs, GenotypeGVCFs, VQSR
	VerifyBamID2	v1.1.3	Contamination check (<5%)
	Hail	v0.2	Variant filtering
SV Calling	Manta	v1.6.0	Primary SV calling (paired-end, split-read, assembly)
	Lumpy	v0.2.13	Auxiliary filtering (read-pair, split-read, read-depth)
	CNVnator	v0.4.1	Auxiliary filtering (read-depth; 150bp window)
SV Filtering/Merging	Paragraph	v2.4a	Re-genotyping short SVs (<200bp)
	Jasmine	v1.1.5	Initial VCF merging (>80% reciprocal overlap), Merging population VCFs for AF database
	Custom Filtering Workflow (Python/bcftools)	v1.10 (bcftools)	Implements overlap logic; GitHub URL https://github.com/xlilab/PathoSV
Repeat Expansion	ExpansionHunter	v4.0.2	Assessed in Ataxia cohort
Annotation/Prioritization	Ensembl VEP	v108	Functional consequence annotation
	CADD	v1.6	Pathogenicity score (SNV/Indel >20 threshold)
	SpliceAI	-	Splicing impact prediction (>0.5 threshold)
	AlphaMissense	-	Missense pathogenicity prediction
	LOFTEE	-	Loss-of-function annotation
	PathoSV (Custom Tool)	-	Transcript-aware SV prioritization; GitHub/Website URLs provided
Key Databases	gnomAD	v3.0 (SNV/Indel)	Population AF (<0.01); Gene constraint
	1000 Genomes Project (1KGP)	Phase 3	Benchmark (NA12878); Background AF (196 East Asian)
	GTEx	v8	Background AF (838 European); Tissue expression (TPMs)
	ClinVar	-	Known variant pathogenicity
	OMIM	-	Gene-disease associations
Visualization	IGV	v2.11.9	Manual variant validation
Statistical Analysis	R	v4.1.2	Fisher's exact test, ROC/AUC analysis

Table S2: Genetic and Molecular Characteristics of Pathogenic Variants Identified in IRD Patients

Family ID	Patient ID (SPID)	Gene	Variant ID[1]	Variant Type[2]	Genomic Location (GRCh38)	Zygosity[3]	Consequence	Inheritance Mode	Retinal TPM	Retinal TDR (Truncated %)	ACMG Criteria Summary[4]
FM2	DT1900044016-1	ABHD12	Var1	SV (INV)	chr20:25313454-25555587	Het	Exon truncation (Exons 1-6)	AR	45.02	99%	PVS1+PS4+PM1+PM2+PM6+PP1+PP4
FM2	DT1900044016-1	ABHD12	Var2	SNV	g.25323332C>T; c.415G>A; p.Gly139Arg	Het	Missense	AR	45.02	65%	PS4+PM1+PM2+PM3+PP1+PP2+PP3+PP4
FM124	RDPYD18030212_A	CHM	Var1	SV (DEL)	chrX:85877526-85879620	Hem	Exon truncation (Exon 13)	XL	17.645	73%	PVS1+PS4+PM1+PM2+PP1+PP4
FM130	RDPYD19022733_A	CHM	Var1	SV (DEL)	chrX:85939182-86023133	Hem	Exon truncation (Exons 3-8)	XL	17.645	80%	PVS1+PS4+PM1+PM2+PP1+PP4
FM185	RDPYD17093654_C	CRB1	Var1	SV (DEL)	chr1:197259028-197272940	Het	Exon truncation (Exon 1)	AR	105.208	86%	PVS1+PS4+PM1+PM2+PP1+PP4
FM185	RDPYD17093654_C	CRB1	Var2	SNV	g.197420998A>G; c.1172-2A>G	Het	Splice acceptor site	AR	105.208	26%	PVS1+PS4+PM2+PP1+PP3+PP4+PP5
FM77	RSZYD18088651_A	DRAM2	Var1	SV (DEL)	chr1:111124590-111125146	Het	Exon truncation (Exon 5)	AR	38.67	58%	PVS1+PS4+PM1+PM2+PP1+PP4
FM77	RSZYD18088651_A	DRAM2	Var2	SNV	g.111118805C>G; c.693G>C; p.Gln231His	Het	Missense / Splice site	AR	38.67	58%	PVS1+PS4+PM1+PM2+PP1+PP2+PP3+PP4
FM95	RDPYD18071407_A	EYS	Var1	SV (DEL)	chr6:64389138-64488837	Het	Exon truncation (Exons 27-28)	AR	51.105	25%	PVS1+PS4+PM1+PM2+PP1+PP4
FM95	RDPYD18071407_A	EYS	Var2	SV (DEL)	chr6:64915646-65010155	Het	Exon truncation (Exons 14-15)	AR	51.105	25%	PVS1+PS4+PM1+PM2+PP1+PP4
FM97	RDPYD18073549_A	EYS	Var1	SV (DEL)	chr6:64620376-64627760	Het	Exon truncation (Exon 23)	AR	51.105	26%	PVS1+PS4+PM1+PM2+PM6+PP1+PP4
FM97	RDPYD18073549_A	EYS	Var2	SNV	g.64230600C>T; c.6416G>A; p.Cys2139Tyr	Het	Missense	AR	51.105	25%	PS4+PM1+PM2+PM6+PP1+PP3+PP4+PP5
FM100	RDPYD18081345_A	EYS	Var1	SV (DEL)	chr6:64911543-64913167	Het	Exon truncation (Exon 16)	AR	51.105	25%	PVS1+PS4+PM1+PM2+PM6+PP1+PP4
FM100	RDPYD18081345_A	EYS	Var2	SNV	g.63721226G>T; c.8805C>A; p.Ter2935*	Het	Nonsense	AR	51.105	25%	PVS1+PS4+PM2+PM6+PP1+PP3+PP4+PP5
FM265	DT2007035500-1	EYS	Var1	SV (DEL)	chr6:64956270-65164613	Het	Exon truncation (Exons 13-14)	AR	51.105	25%	PVS1+PS4+PM1+PM2+PM6+PP1+PP4
FM265	DT2007035500-1	EYS	Var2	SNV	g.64230600C>T; c.6416G>A; p.Cys2139Tyr	Het	Missense	AR	51.105	25%	PS4+PM1+PM2+PM6+PP1+PP3+PP4+PP5
FM13	RDPYD18083944_A	PDE6B	Var1	SV (DEL)	chr4:633534-637421	Hom	Exon truncation (Exons 2-3)	AR	483.789	53%	PVS1+PS4+PM1+PM2+PM6+PP1+PP4
FM105	RSZYD17061140_A	PDE6B	Var1	SV (DEL)	chr4:633534-637421	Hom	Exon truncation (Exons 2-3)	AR	483.789	53%	PVS1+PS4+PM1+PM2+PM6+PP1+PP4
FM112	RDPYD17093636_C	PROM1	Var1	SV (DEL)	chr4:15992516-15997089	Het	Exon truncation (Exon 15)	AD	575.619	61%	PVS1+PS4+PM1+PM2+PP1+PP4
FM157	D2004011113	PROM1	Var1	SV (DEL)	chr4:16012199-16038605	Het	Exon truncation (Exons 3-10)	AR	575.619	64%	PVS1+PS4+PM1+PM2+PP1+PP4
FM157	D2004011113	PROM1	Var2	SV (DEL)	chr4:16031002-16041704	Het	Exon truncation (Exons 2-4)	AR	575.619	64%	PVS1+PS4+PM1+PM2+PP1+PP4
FM289	DT1900000549-1	PROM1	Var1	SV (DEL)	chr4:15992516-15997089	Hom	Exon truncation (Exon 15)	AR	575.619	61%	PVS1+PS4+PM1+PM2+PP1+PP4
FM297	DT2005020102-1	PROM1	Var1	SV (DEL)	chr4:15992516-15997089	Het	Exon truncation (Exon 15)	AR	575.619	61%	PVS1+PS4+PM2+PP1+PP4
FM297	DT2005020102-1	PROM1	Var2	SNV	g.15998382T>A; c.1682+3A>T	Het	Splice donor site	AR	575.619	61%	PVS1+PS4+PM2+PM6+PP1+PP3+PP4
FM52	RDPYD18125285_A	PRPF31	Var1	SV (DEL)	chr19:54064664-54133055	Het	Whole gene deletion	AD	21.33	100%	PVS1+PS4+PM1+PM2+PP1+PP4
FM96	RDPYD18073535_A	PRPF31	Var1	SV (DEL)	chr19:54118226-54122901	Het	Exon truncation (Exons 2-5)	AD	21.33	100%	PVS1+PS4+PM1+PM2+PM6+PP4
FM129	RDPYD19020035_A	PRPF31	Var1	SV (DEL)	chr19:54099322-54133113	Het	Whole gene deletion	AD	21.33	100%	PVS1+PS4+PM1+PM2+PM6+PP1+PP4
FM151	D2004011111	PRPF31	Var1	SV (DEL)	chr19:53996498-54132343	Het	Whole gene deletion	AD	21.33	100%	PVS1+PS4+PM1+PM2+PM6+PP1+PP4
FM78	RSZYD18088676_A	RS1	Var1	SV (DEL)	chrX:18644599-18650206	Hem	Exon truncation (Exons 4-5)	XL	305.285	100%	PVS1+PS4+PM1+PM2+PM6+PP1+PP4
FM65	RDPYD18072867_A	TULP1	Var1	SV (DEL)	chr6:35502609-35506996	Het	Exon truncation (Exons 8-12)	AR	316.001	96%	PVS1+PS4+PM1+PM2+PP1+PP4
FM65	RDPYD18072867_A	TULP1	Var2	SNV	g.35512178A>C; c.190+2T>G	Het	Splice donor site	AR	316.001	59%	PVS1+PS4+PM2+PM6+PP1+PP3+PP4
FM110	RDPYD17082158_A	USH2A	Var1	SV (DEL)	chr1:216072986-216073478	Het	Exon truncation (Exon 28)	AR	28.616	97%	PVS1+PS4+PM1+PM2+PM6+PP1+PP4
FM110	RDPYD17082158_A	USH2A	Var2	SNV	g.215674572T>C; c.13339A>G; p.Met4447Val	Het	Missense	AR	28.616	97%	PS4+PM1+PM2+PM6+PP1+PP3+PP4+PP5

Footnotes:

[1] Var1, Var2 denote variants found in compound heterozygous state.

[2] SV=Structural Variant; SNV=Single Nucleotide Variant; DEL=Deletion; INV=Inversion.

[3] Zygosity: Het=Heterozygous; Hom=Homozygous; Hem=Hemizygous.

[4] ACMG Criteria Summary lists evidence codes supporting pathogenicity classification.

Table S3: Clinical Phenotypes of IRD Patients with Identified Pathogenic Variants

Family ID	Patient ID (SPID)	Gene	Clinical Diagnosis	Inheritance Mode	Key Ocular Features
FM2	DT1900044016-1	ABHD12	Retinitis Pigmentosa (RP)	AR	RP features
FM124	RDPYD18030212_A	CHM	Choroideremia	XL	Advanced chorioretinal atrophy, night blindness, visual field constriction
FM130	RDPYD19022733_A	CHM	Choroideremia	XL	Advanced chorioretinal atrophy, night blindness, visual field constriction
FM185	RDPYD17093654_C	CRB1	Retinitis Pigmentosa (RP)	AR	Consistent with RP
FM77	RSZYD18088651_A	DRAM2	Cone-Rod Dystrophy (CRD)	AR	Decreased VA, abnormal color vision, macular atrophy, ERG: cone > rod dysfunction
FM95	RDPYD18071407_A	EYS	Retinitis Pigmentosa (RP)	AR	Consistent with RP
FM97	RDPYD18073549_A	EYS	Retinitis Pigmentosa (RP)	AR	Consistent with RP
FM100	RDPYD18081345_A	EYS	Retinitis Pigmentosa (RP)	AR	Consistent with RP
FM265	DT2007035500-1	EYS	Retinitis Pigmentosa (RP)	AR	Consistent with RP
FM13	RDPYD18083944_A	PDE6B	Retinitis Pigmentosa (RP)	AR	Consistent with RP
FM105	RSZYD17061140_A	PDE6B	Retinitis Pigmentosa (RP)	AR	Consistent with RP
FM112	RDPYD17093636_C	PROM1	Stargardt (STGD)-like Disease	AD	Macular RPE atrophy ("bull's eye"/"beaten bronze"), yellow pisciform flecks, outer retinal structural loss
FM157	D2004011113	PROM1	Retinitis Pigmentosa (RP)	AR	Consistent with RP
FM289	DT1900000549-1	PROM1	Retinitis Pigmentosa (RP)	AR	Consistent with RP
FM297	DT2005020102-1	PROM1	Retinitis Pigmentosa (RP)	AR	Consistent with RP
FM52	RDPYD18125285_A	PRPF31	Retinitis Pigmentosa (RP)	AD	Consistent with RP
FM96	RDPYD18073535_A	PRPF31	Retinitis Pigmentosa (RP)	AD	Consistent with RP
FM129	RDPYD19020035_A	PRPF31	Retinitis Pigmentosa (RP)	AD	Consistent with RP
FM151	D2004011111	PRPF31	Retinitis Pigmentosa (RP)	AD	Consistent with RP
FM78	RSZYD18088676_A	RS1	Retinoschisis	XL	Consistent with Retinoschisis
FM65	RDPYD18072867_A	TULP1	Retinitis Pigmentosa (RP)	AR	Consistent with RP
FM110	RDPYD17082158_A	USH2A	Retinitis Pigmentosa (RP)	AR	Night blindness, reduced visual field, waxy optic disc, vessel attenuation, bone-spicule pigment deposits, retinal atrophy, absent ERG

Footnotes:
Abbreviations: RP=Retinitis Pigmentosa; CRD=Cone-Rod Dystrophy; STGD=Stargardt Disease; AR=Autosomal Recessive; AD=Autosomal Dominant; XL=X-linked; NR=Not Reported (specific age not available in summary data, cohort average onset was 15.0 years, range 2-50); VA=Visual Acuity; ERG=Electroretinography; RPE=Retinal Pigment Epithelium.

Table S4: Genetic and Molecular Characteristics of Structural Variants Identified in HA Patients

Patient ID (SPID)	Gene Symbol	Chr	Start Pos (GRCh38)	End Pos (GRCh38)	SV Type	SV Length (bp)	Consequence	Background AF[1]	OMIM Inheritance[2]	Sum Truncated TPM[3]	Sum All TPM[3]	TDR (Truncated %)[4]	ACMG Criteria Summary[5]
M13798	ITPR1	3	4493139	4775085	DEL	281946	Exon truncation (Exons 1-43)	T0	AD, AR	63.6	94.4	67%	PVS1+PM2+PP3
M33532	PABPN1	14	23322276	23333319	DUP	11043	Exon truncation (Exons 2-6)	T0	AD	298.3	298.3	100%	PVS1+PM2+PP3
M634	EIF2AK2	2	37111866	37117545	DEL	5679	Exon truncation (Exons 14)	T0	AD, AR	6.4	6.6	97%	PVS1+PM2+PP3
M20144	TMEM63A	1	225848585	225865586	DEL	17001	Exon truncation (Exons 1-22)	T0	AD	13.1	14.9	88%	PVS1+PM2+PP3
M26276	TMEM63A	1	225853176	225857046	DEL	3870	Exon truncation (Exons 16-19)	T0	AD	9.3	14.9	63%	PVS1+PM2+PP3
M39291	GABRB3	15	26716752	26717128	DEL	376	Exon truncation (Exons 1)	T0	AD	8.5	16.3	52%	PVS1+PM2+PP3
M6655	SPG7	16	89512072	89533328	DEL	21256	Exon truncation (Exons 3-9)	T0	AD, AR	68.8	131.4	52%	PVS1+PM2+PP3
M39278	SPAST	2	32135173	32177991	DEL	42818	Exon truncation (Exons 10-17)	T0	AD	5.8	5.8	100%	PVS1+PM2+PP3
M16497	SPAST	2	32154387	32163775	DEL	9388	Exon truncation (Exons 17)	T0	AD	5.8	5.8	100%	PVS1+PM2+PP3
M30463	SLC20A2	8	42416754	42417430	DUP	676	Exon truncation (Exons 11)	T0	AD	36.4	49.8	73%	PVS1+PM2+PP3

Footnotes:

[1] Background AF: T0 indicates the variant was not observed (Allele Frequency = 0) in the background population database used.

[2] OMIM Inheritance: AD = Autosomal Dominant; AR = Autosomal Recessive; XL = X-linked. This column indicates the known inheritance pattern(s) for diseases associated with the gene.

[3] TPM: Transcripts Per Million. Data indicates the sum of expression levels (TPM) for transcripts predicted to be truncated by the SV vs. all transcripts of the gene, and the resulting percentage.

[4] Truncated percentage calculated from provided TPM values (Sum Truncated / Sum All). High percentages suggest most or all major transcripts are affected.

[5] ACMG Criteria Summary lists evidence codes supporting pathogenicity classification.

Table S5: Clinical Phenotypes and Assessments of HA Patients with Identified Structural Variants

Patient ID (SPID)	Age at Onset (years)	Age at Assessment (years)	Clinical Diagnosis (OMIM #, Name)	Key Clinical Features at Assessment	Progression	Family History	SARA	ICARS
M13798	26	33	606658, SCA15; 117360, SCA29	Ataxic gait (drunk-like), dysarthria, recent dysphagia/choking, normal eye movements/strength, impaired coordination (FNT+)	Gradual worsening	Mother (tremor, unstable gait); Brother (onset 22y)	6	10
M33532	37	47	164300, OPMD	R leg weakness, gait instability, lifelong dysarthria, visual impairment, normal eye movements, R leg hypertonia, hyperreflexia, wide-based gait, impaired coordination (FNT+, HKS+, Romberg+)	Gradual worsening	Brother (44y, similar symptoms >10y)	7.5	24
M634	44	45	619687, Dystonia 33; 618877, Leukoencephalopathy	Dysarthria, gait instability (worsened recently), limb stiffness, clumsiness, weight loss, normal eye movements, hyperreflexia UE. CT: Cerebellar atrophy. EMG: Myopathic.	Gradual worsening	7 affected relatives, 3 deceased with progressive symptoms (dysarthria, atrophy, ptosis, spasm, tremor, death).	NA	NA
M20144	7	9	618688, Hypomyelinating Leukodystrophy 19	Gait instability (toddling), dysarthria, slight visual decline, occasional leg pain, normal eye movements/strength/tone, hyperreflexia, impaired coordination (FNT+, Romberg+). MRI: Cerebellar atrophy.	Assessed early in course	None reported (Sporadic)	11	33
M26276	0 (Congenital/Early onset)	24	618688, Hypomyelinating Leukodystrophy 19	Long history (>20y) gait instability, dysarthria, developmental delay (speech 3y, walking 4y), easy falls, limited eye movements (upgaze, adduction, abduction), LE hypertonia, hyperreflexia LE, wide-based gait, impaired coordination (FNT+, HKS+, Romberg+), impaired position sense.	Stable/Slowly progressive	Parents consanguineous, no other affected.	11.5	36
M39291	12	29	617113, DEE43	Gait instability (cannot walk straight, avoids stairs), worsening, frequent falls (last 7y), constipation, horizontal nystagmus, hypotonia, areflexia, toddling gait, impaired coordination (HKS+, Romberg+), impaired position sense, Chaddock sign +.	Gradual worsening	None reported (Sporadic)	7.5	26
M6655	32	40	607259, SPG7	Gait instability, dysarthria (onset 36y), occasional choking, normal eye movements/strength/tone, hyperreflexia LE, wide-based gait, impaired coordination (FNT+, HKS+), normal sensation, negative pathological signs.	Assessed mid-course	None reported (Sporadic)	7	18
M39278	33	38	182601, SPG4	Gait instability (spastic), occasional dizziness, difficulty turning, horizontal/vertical nystagmus, LE hypertonia, hyperreflexia LE, spastic gait, positive Romberg (eyes closed), bilateral Babinski +.	Gradual worsening	Father (onset 50+), Paternal Grandfather (onset 50-60s)	5	14
M16497	50	70	182601, SPG4	Long history (>20y) dragging feet, progressive difficulty walking/stairs (needs cane recently), constipation, no nystagmus noted, sensation normal.	Gradual worsening	Third son (35y, gait unstable 2y)	9	30
M30463	10	32 (1st visit) / 41 (2nd visit)	213600, IBGC1	R leg limp/difficulty stairs (onset 10y), R hand tremor, R groin pain on walking (onset ~27y). 2nd visit: Slow progression, clumsiness (onset ~26y). Dysarthria, horizontal nystagmus, R sided hypertonia, hyperreflexia/pathological signs. Wide-based gait, impaired coordination (Romberg+, R sided dysdiadochokinesia). MRI normal.	Gradual worsening	Father and Paternal Grandfather similar symptoms	5 (1st) / 6.5 (2nd)	7 (1st) / 22 (2nd)

Footnotes:
Abbreviations: SCA=Spinocerebellar Ataxia; OPMD=Oculopharyngeal Muscular Dystrophy; DEE=Developmental and Epileptic Encephalopathy; SPG=Spastic Paraplegia; MGA=Methylglutaconic Aciduria; IBGC=Idiopathic Basal Ganglia Calcification; FNT=Finger-Nose Test; HKS=Heel-Knee-Shin Test; UE=Upper Extremities; LE=Lower Extremities; SARA=Scale for the Assessment and Rating of Ataxia; ICARS=International Cooperative Ataxia Rating Scale.
